# Dose–response of tDCS effects on motor learning and cortical excitability: A preregistered study

**DOI:** 10.1162/imag_a_00431

**Published:** 2025-01-15

**Authors:** Gavin Hsu, Zhenous Hadi Jafari, Abdelrahman Ahmed, Dylan J. Edwards, Leonardo G. Cohen, Lucas C. Parra

**Affiliations:** Department of Biomedical Engineering, The City College of New York, New York, NY, United States; Moss Rehabilitation Research Institute, Elkins Park, PA, United States; School of Medical and Health Sciences, and Exercise Medicine Research Institute, Edith Cowan University, Joondalup, WA, Australia; National Institute of Neurological Disorders and Stroke, National Institutes of Health, Bethesda, MD, United States

**Keywords:** transcranial electric stimulation, neuromodulation, motor task, TMS, EEG, plasticity

## Abstract

Multiple studies have demonstrated that transcranial direct current stimulation (tDCS) of the primary motor cortex (M1) can influence corticospinal excitability and motor skill acquisition. However, the evidence for these effects is inconsistent, and a common neural substrate for these effects has not been directly demonstrated. To address this, we hypothesized that higher tDCS intensities would produce more robust effects, and uncover their relationship. In this preregistered study, 120 participants engaged in a motor skill learning task while receiving tDCS with posterior-to-anterior currents through M1. We employed a double-blind, between-subjects design, with groups of 4 mA, 6 mA, or sham stimulation, while ensuring balanced groups in terms of typing speed. Cortical excitability was assessed via motor-evoked potentials (MEPs) and TMS-evoked potentials (TEPs) before and after motor skill learning with concurrent tDCS. tDCS at these higher intensities was well tolerated, and motor learning correlated with pretraining typing speed.*Planned analyses*found no dose–response effect of tDCS on motor skill performance or MEP amplitude. This suggests that, under our experimental conditions, tDCS did not significantly modulate motor skill learning or corticospinal excitability. Furthermore, there was no correlation between motor performance and MEP, and thus no evidence for a common neural substrate.*Exploratory analyses*found an increase in MEP and TEP amplitudes following the sequence learning task. Motor skill gains positively correlated with TEP changes over the stimulated M1, which were more negative with increasing tDCS intensity. The effects of tDCS on motor skill learning and MEPs, if they exist, may require particular experimental conditions that have not been tested here. Preregistration:https://osf.io/jyuev(in-principle acceptance: 2024/06/05)

## 1 Introduction

Transcranial direct current stimulation (tDCS) has been widely studied as an intervention to modulate cortical brain activity.*In vitro*animal studies have shown that electric fields induced by electrical stimulation can polarize the somatic membrane of neurons (Bikson et al., 2004), which can in turn increase or decrease neuronal firing rates (Farahani et al., 2023;Reato et al., 2010). Changes in cortical excitability have been observed*in vivo*in human experiments that target the primary motor cortex (M1;Nitsche & Paulus, 2000). These experiments measured motor-evoked potentials (MEPs) in a hand muscle elicited by transcranial magnetic stimulation (TMS) over the motor cortex. This corticospinal excitability is increased following “anodal” tDCS (with inward current flow at the region of interest) and a decrease following “cathodal” tDCS (with outward current;Nitsche & Paulus, 2000). tDCS effects on the motor system can also be assessed behaviorally, as seen in some experiments that found that anodal stimulation applied over M1 enhanced motor skill learning (Galea & Celnik, 2009;Nitsche et al., 2003;Stagg, Jayaram, et al., 2011). It is widely believed that the two phenomena have a common physiological substrate, such as a modulation of synaptic efficacy involved in motor skill learning (Floyer-Lea et al., 2006;Karni et al., 1995;Kawai et al., 2015;Kolasinski et al., 2019;Muellbacher et al., 2002;Olivo et al., 2022;Pascual-Leone et al., 1995;Penhune & Doyon, 2005;Penhune & Steele, 2012;Stagg, Bachtiar, et al., 2011;Ziemann et al., 2004). While there is ample evidence that various interventions with electric and magnetic stimulation can modulate plasticity in M1 (Karabanov et al., 2015), human behavioral studies have not shown a consistent effect of motor skill learning on MEPs (Cao et al., 2022;Christiansen et al., 2018;Classen et al., 1998;Garry et al., 2004;Hirano et al., 2017;Koeneke et al., 2006;Miyaguchi et al., 2019;Muellbacher et al., 2001,^2002^;Rosenkranz et al., 2007;Smyth et al., 2010). Studies on the effects of tDCS on motor skill learning have given mixed results (Ambrus et al., 2016;Buch et al., 2017;Küper et al., 2019;Nguemeni et al., 2021), and there is skepticism around reproducibility of MEP effects of tDCS (Horvath et al., 2015,^2016^). Some null results of tDCS on MEP may be explained as reversal of effects at increasing intensities in the range of 1 to 2 mA (Batsikadze et al., 2013;Hassanzahraee et al., 2020) leading to a “no man’s land” at intermediate intensities (Stagg et al., 2018). Especially at lower intensities (1 mA), null results on learning behavior and excitability (Wiltshire & Watkins, 2020) have been found along with conflicting findings at 2 mA (Fiori et al., 2014;M. N. Wong et al., 2019). We hypothesized a monotonic effect of tDCS where higher intensities produce more prominent effects, but remained open to the possibility of a reversal of the effects on MEP or motor skill learning. If the dose–response of MEP was to reverse with increasing tDCS intensity, but was monotonic for skill learning (or vice versa), it would have been more difficult to reconcile this with a common physiological substrate.

Our hypothesis of an improvement in the consistency of*in vivo*human tDCS effects with increasing tDCS intensity was based on our earlier*in vitro*findings of a monotonic dose–response of field strength in synaptic plasticity (Kronberg et al., 2020;Sharma et al., 2022). However,*in vitro*current densities are much higher than those applied in human studies, so dose effects may not translate (Esmaeilpour et al., 2018;Farahani et al., 2021). Animal models and*in vitro*experiments typically apply 5 V/m or higher, compared with the relatively low estimated fields in humans of less than 1 V/m at 2 mA (Liu et al., 2018). Although effects have been demonstrated*in vitro*under electric fields as small as 0.2 V/m with alternating currents (Reato et al., 2010), the dose–response of direct currents below 2.5 V/m has not been investigated. Furthermore, since current intensities in most of the human studies literature were limited at 2 mA, characteristics of tDCS effects at higher levels are largely unknown (Esmaeilpour et al., 2018). We were, therefore, motivated to investigate the dose–response of tDCS effects above 2 mA. Within the scope of this study, we focused on anodal tDCS, as effects of cathodal stimulation are asymmetrical (Agboada et al., 2019;Batsikadze et al., 2013;Kronberg et al., 2020;Lafon et al., 2017;Mosayebi Samani et al., 2019). There is evidence of a positive effect of anodal tDCS intensity on motor learning performance at intensities up to 1.5 mA (Cuypers et al., 2013). This follows a similar monotonic dose–response at up to 2 mA in cerebral blood flow (CBF;Jamil et al., 2020;Zheng et al., 2011), but contradicts other studies that have consistently found a nonlinear relationship with MEP amplitude at up to 2 mA (Bastani & Jaberzadeh, 2013;Chew et al., 2015;Hassanzahraee et al., 2020;Jamil et al., 2017;Kidgell et al., 2013;Tremblay et al., 2016). Notably, all but one of these works cited (Tremblay et al., 2016) did not employ neuronavigation, which is generally thought to improve TMS accuracy (Sack et al., 2009) and, more importantly, targeting stability (Bashir et al., 2011). It is less clear whether a similar nonlinear effect continues above 2 mA for behavioral learning effects.Agboada et al. (2019)found that 1, 2, and 3 mA all increased MEP amplitudes, but without significant differences across the intensities. However,Shinde et al. (2021)demonstrated a monotonic dose–response of tDCS across 0.1, 2, and 4 mA in both CBF and behavioral outcomes in the region of interest under the anode. We also found a positive behavioral effect on motor skill learning at 4 mA (Hsu et al., 2023), but those results with a new electrode configuration remain to be replicated. In total, there is conflicting literature on the dose–response of tDCS on both motor skill learning and MEPs. To address this, we tested the dose–response of tDCS at up to 6 mA, which was made possible by the use of HD electrodes to mitigate sensation by spreading out current intensity on the scalp (Hsu et al., 2023).

The conflicting dose–response relationships call into question, additionally, the hypothesis that behavioral and MEP effects have the same physiological substrate. Indeed, changes in M1 activity during and following motor learning as observed with neuroimaging (Muellbacher et al., 2001,^2002^) are not reflected consistently in MEP amplitude changes. While simple repetitive movements can affect M1 excitability (Classen et al., 1998;Koeneke et al., 2006;Muellbacher et al., 2001,^2002^;Rosenkranz et al., 2007), studies have found that sequence learning tasks do not (Cao et al., 2022;Hirano et al., 2017). MEP changes resulting from use-dependent plasticity during motor practice are evidently linked to GABA inhibition (Bütefisch et al., 2000;Ziemann et al., 2001). Although GABA concentration can be reduced by tDCS (Stagg et al., 2009), motor learning with tDCS does not appear to yield a consistent correlation between MEP amplitude and GABA synaptic activity or motor skill acquisition (Amadi et al., 2015;Ambrus et al., 2016). Time may be yet another factor, as weeks of skill training have been shown to increase excitability (Pascual-Leone et al., 1995), in contrast with strength training (Jensen et al., 2005). Even in the absence of skill learning, tDCS effects on MEP amplitudes are evidently state dependent, varied by M1 activation with a simple motor task (Antal et al., 2007;Pellicciari et al., 2013). We measured both performance and excitability changes, and the presence or absence of a correlation between them may help address some of these uncertainties around what neural pathways are modulated by tDCS.

We were motivated by the causal model of mechanistic effects presented inFigure 1. Here the effect of learning and electric fields generated by tDCS interact to affect both motor performance and MEP amplitude. The factor mediating this interaction (white box) is commonly thought to be synaptic plasticity, but the current experiment does not measure this directly, so all we are assuming is that there is a common physiological substrate. Based on this model, we hypothesized a monotonic relationship between performance on a motor skill learning task and tDCS intensity (H1), and a monotonic relationship between MEP change and tDCS intensity (H2). We hypothesized that when controlled for tDCS intensity, motor learning task performance is positively correlated with change in MEP amplitude (H3). As an exploratory objective, we also observed changes in cortical excitability as measured through TMS-evoked potentials (TEPs) using electroencephalography (EEG), as opposed to corticospinal excitability measured in MEPs (Ferreri & Rossini, 2013;Hernandez-Pavon et al., 2023;Ilmoniemi et al., 1997;Tremblay et al., 2019).

**Fig. 1.**
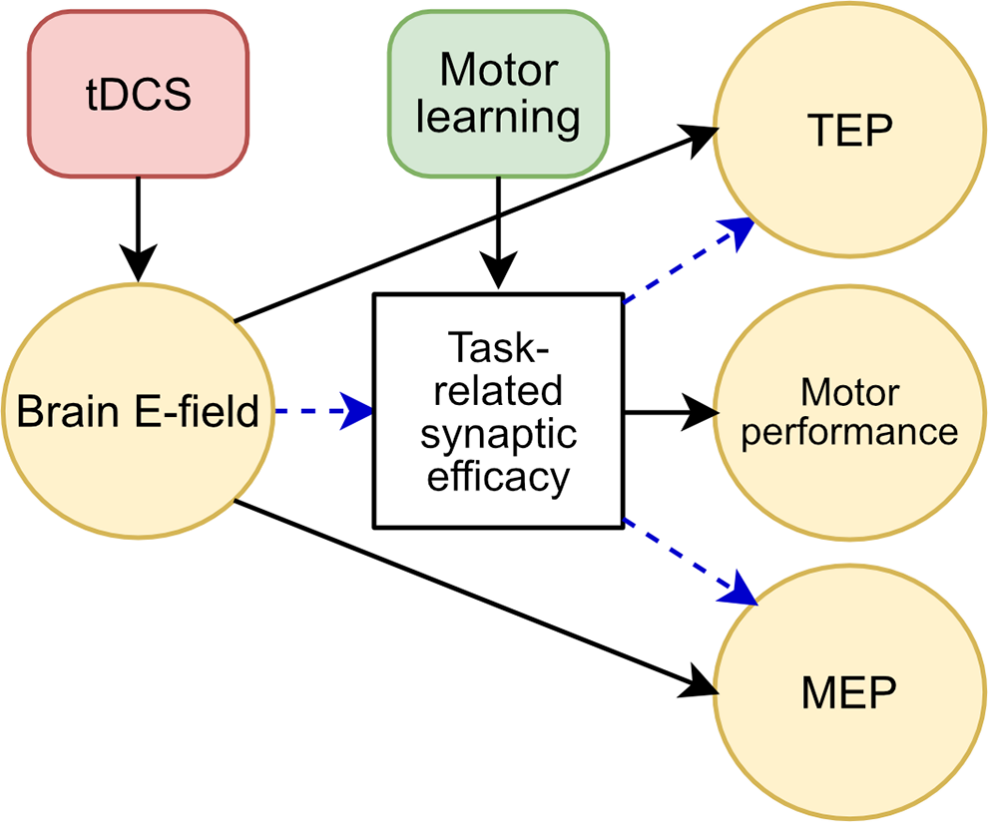
A proposed model of tDCS action and interaction with motor skill learning in human studies. We experimentally control tDCS intensity (red) to determine its effect on experimental outcome variables (yellow). Solid lines indicate established causal effects. For instance, motor skill learning (green) is thought to causally affect motor performance via changes in task-related synaptic efficacy in M1. Similarly, transcranial currents produce electric fields in the brain that are thought to modulate MEP and TEP magnitudes. Dashed lines indicate causal mechanisms hypothesized here that are less well established. For instance, while there is clear*in vitro*evidence that electric fields can modulate synaptic efficacy, it is not clear that this is the mechanism by which tDCS affects performance outcomes in motor skill learning. We previously postulated an interaction between behavioral training and electric fields to affect synaptic efficacy (i.e., both are required) (Kronberg et al., 2020). It is often assumed that electric fields affect MEPs via a modulation of synaptic efficacy, but evidence for this is conflicting, as discussed in the main text.

The use of TEPs as an indicator of plasticity and excitability changes following tDCS is a relatively recent development. Like MEPs, TEP amplitudes have been found to increase after anodal tDCS and even correlate with MEP amplitudes (Ahn & Fröhlich, 2021;Pellicciari et al., 2013). More recently,Mosayebi-Samani et al. (2023)found a nonlinear dose–response of TEPs to cathodal tDCS over M1 at up to 2.1 mA. Whereas 1.4 mA tDCS resulted in an increase in early TEP amplitudes but no effect on MEP amplitudes, 0.7 and 2.1 mA tDCS resulted in decreased TEP amplitudes and MEP amplitudes, with significant positive correlations. We collected similar measurements of cortical excitability and determined whether TEPs can capture the synaptic changes occurring during motor learning with tDCS. Due to a lack of standardization of TMS-EEG techniques and analysis (Hernandez-Pavon et al., 2023), we did not commit to a preplanned analysis pipeline for TEP data. Based on the results from the few existing studies with tDCS, we hypothesized a monotonic relationship between tDCS intensity and TEP amplitude. We also expected positive correlations between TEP amplitudes and performance as well as MEP amplitudes.

Finally, this study served as a partial replication of our previous study at 4 mA where we also tested whether tDCS effects outlasted the period of stimulation and whether they were specific to the stimulated hemisphere and task. The experimental design, therefore, closely matched the previous study (Hsu et al., 2023).

## 2 Methods

### 2.1 Experimental design

Subjects were algorithmically assigned to one of three groups, each corresponding to a different stimulation condition. At the beginning of the experiment, each subject completed a typing test to assess baseline typing speed that served to control for intersubject variability. Group assignment was selected at random while minimizing variation in typing speed across groups. The study was double blinded by delegating different tasks between two researchers. One researcher was responsible for monitoring the group assignments, as well as setting the stimulation intensity on the stimulator and operating the stimulator. The stimulator settings were obscured from the second researcher, who prepared subjects for tDCS and explained the behavioral task to the subject. The second researcher, who was blinded to the tDCS condition, also conducted the TMS and MEP data collection procedures before and after tDCS, while the first researcher assisted. Code for data preprocessing and analysis was written and tested without knowledge of individual subjects’ stimulation condition, until the final reporting of the outcome variables listed in the Analysis Plan below.

### 2.2 Subjects

This study was conducted on healthy right-handed adults, consisting primarily of students recruited on the campuses of the City University of New York. Informed written consent was obtained from all participants for inclusion in the study, under approval by the City University of New York Institutional Review Board. Prospective participants were screened and excluded if there were any contraindications to TMS and tDCS. These included a history of seizures, fainting, or head trauma; pregnancy; chronic headaches, nausea, and drowsiness; metal implants in the head; wounds or chronic skin disorders on the scalp; thick and tightly braided hairstyles; weaves and wigs; and any headgear that cannot be removed. For the purpose of this study, other exclusion criteria included left handedness, neurological or psychiatric disorders, taking nervous system medication, severe visual impairment, chronic abuse of psychoactive substances, and disability of the left upper extremity. Subjects with experience of tDCS were excluded from the study to facilitate blinding. Subjects were asked to not consume alcohol or other psychoactive substances (except caffeine) on the day of the experiment. Individuals with experience in playing musical instruments involving sequential finger movements were also excluded from the study, unless they had stopped playing for a number of years exceeding the years of experience.

A total of 130 participants (sex: 73 female and 57 male, age: mean ± SD = 23.3 ± 5.86 years, range 18–61 years) were recruited for the study. We initially recruited 121 participants and 1 participant did not complete the procedures due to an adverse reaction to TMS. Following quality control checks of the motor performance data, we recruited 9 more participants to replace excluded samples such that there were 40 valid motor performance samples in each group.

### 2.3 Procedures

Procedures followed the timeline outlined inFigure 2c. Up to 2 experimental sessions were conducted per day, starting either at 9:00 AM or 1:00 PM.

**Fig. 2.**
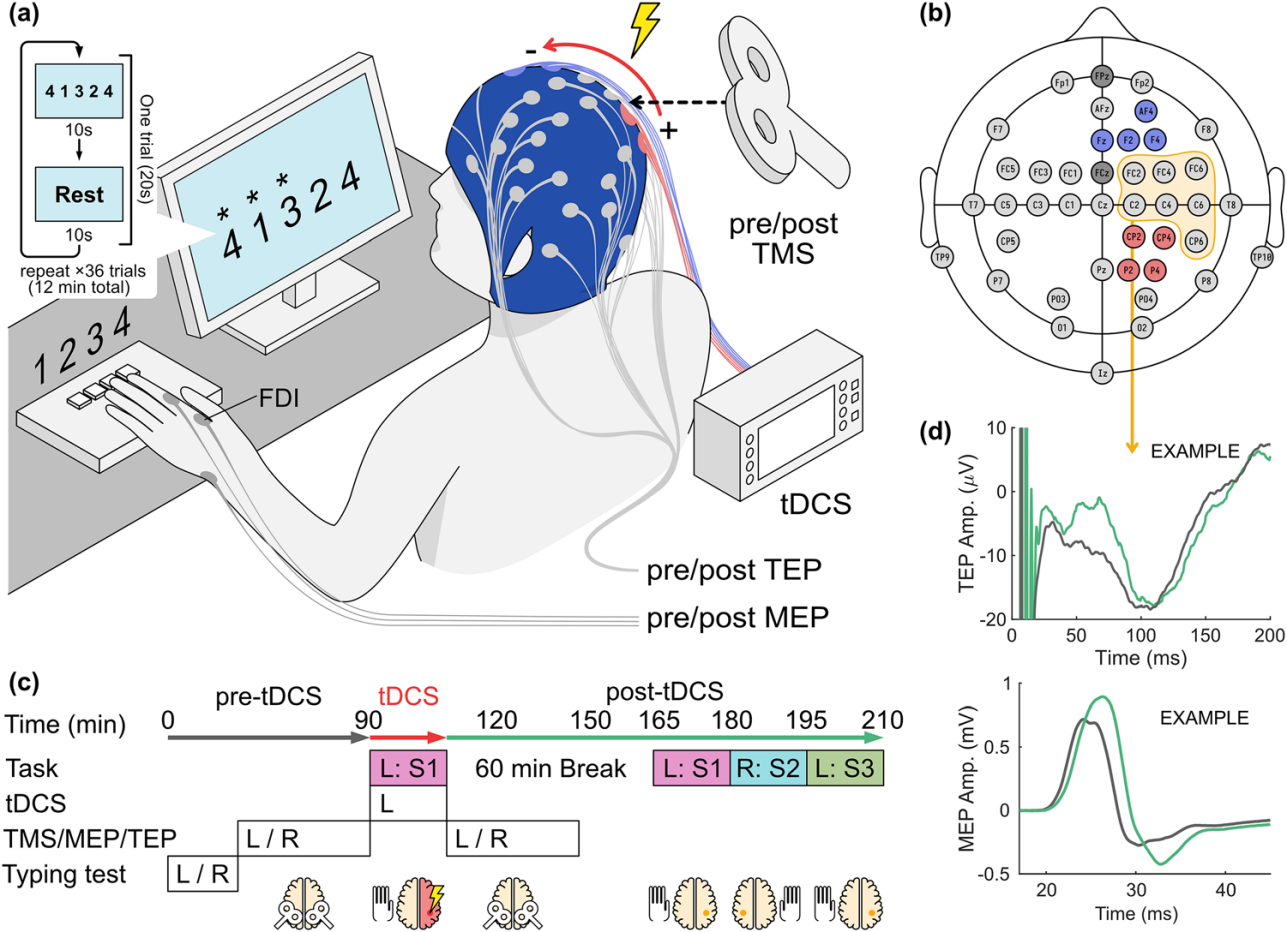
Experimental setup and timeline. (a) The subject received “anodal” tDCS over the right M1 during 12 min of motor sequence learning (Task) performed using the left (nondominant) hand. A monitor displayed a five-element sequence for the subjects to replicate by pressing four buttons with their left-hand fingers. Each finger was assigned one key labeled with a corresponding number. TMS was applied pre- and post-tDCS to measure MEPs and TEPs. (b) A custom electrode cap was used to both deliver tDCS and record EEG. Electrical stimulation was spread out across four parietal “anodes” (red) and four frontal “cathodes” (blue) with currents flowing from posterior to anterior direction (Hsu et al., 2023). Thirty-two EEG channels (light gray) were distributed symmetrically across the midline and around the tDCS electrode locations. (c) The subject first completed a baseline typing task to determine group placement based on baseline typing speed. TMS-evoked MEPs were measured from both the left and then the right FDI before and after the initial sequence learning and tDCS. At 60 min after the end of tDCS and initial task training, the subject repeated the sequence learning task to test whether learning effects are lasting (R:S1), specific to hemisphere (R:S2), and specific to the trained sequence (L:S3). (d) Local TEPs were sampled from the electrodes close to the hand area of M1, approximately at the C3 and C4 locations. Pre-/postamplitudes were compared, as shown in the example (gray/green, respectively). Likewise, pre-/post-MEP amplitudes from the same TMS trials were compared, as shown in the example.

#### 2.3.1 Typing test and group assignments

Touch typing proficiency has previously been associated with manual dexterity, visuomotor coordination, and motor speed (Austin et al., 2011;Chwirka et al., 2002;McGlashan et al., 2017). We found in a pilot study that there is a considerable positive correlation (*r*= 0.58) between baseline typing speed and baseline sequence learning performance (see below: “Predicting motor learning ability”). Here we used a baseline typing task to measure typing speed. This was used to balance group assignments and as a covariate to control for variance across subjects. This task is broadly recognizable as most adults are already experienced to some extent, making it easy to administer with minimal instruction. It is also sufficiently different from the main task, so as to minimize interference with the actual sequence learning task.

The typing test involved correctly typing a series of 63 common English words as quickly as possible using both hands. Either multiple or single finger typing was allowed. Because typing with multiple fingers is generally faster than typing with single fingers, typing speed was expected to capture multifinger dexterity similar to that required in the sequence learning task. The words appeared in a sequence of 22 characters (including spaces) on a screen in front of the subject, scrolling leftward as the subject correctly typed out characters. A stationary cursor highlighted the leftmost character to be typed next. The task continued until all characters were pressed correctly. There was no time limit on this task, but we estimated that it can typically be completed in approximately 1 min. The results from this baseline task were evaluated using a script in MATLAB (MathWorks, Natick, MA) that also managed group assignments. Typing speed was measured as words per minute (WPM). Initially, the first three subjects were sequentially assigned to the three groups. Subsequent subjects were then sorted by the grouping that resulted in the smallest summed variance across groups, as described bySella et al. (2021). This form of “covariate-adaptive” method of minimization, originally conceived byTaves (1974)andPocock and Simon (1975), can help limit imbalances in randomized controlled trials (Scott et al., 2002;Sella et al., 2021).

#### 2.3.2 MEP measurement

MEPs were measured immediately before and after tDCS (Fig. 2). The subjects were seated on a chair with an armrest and asked to relax their arm. Electromyography (EMG) recordings were collected using an eego mini EMG amplifier (ANT Neuro, Hengelo, Netherlands) at either 500 Hz or 2 kHz from the left first dorsal interosseous (FDI) muscle through foam electrodes and triggered by single-pulse TMS using a MEGA-TMS system (Soterix Medical, Woodbridge, NJ) with a standard figure-of-eight coil (100 mm diameter). TMS pulses were delivered to the M1 region representing the FDI, which was located using a visor2 neuronavigation system (ANT Neuro, Hengelo, Netherlands). Starting from the C4 electrode location on the 10–10 system, we searched using 1 cm steps in the coronal and sagittal planes for the FDI “hotspot” that yielded the highest MEP amplitude at a given output level. The increments were lowered to less than 5 mm as we approached the “hotspot”, continuing until the MEP amplitude appeared to stop increasing. The coil was oriented at a 45 degree angle toward the midline. Resting motor threshold (RMT) was determined as the minimal output level necessary to produce an MEP in at least 6 out of 10 pulses. Sixty pulses were delivered at 120% RMT, with at least 5 s between pulses. After measurement of the left FDI MEPs, the same procedures were applied to the right FDI, stimulating over the left M1, near the C3 electrode location. The same TMS output level was used before and after tDCS and training. Neuronavigation was employed to ensure precise placement of the coil across multiple trials and sessions such that the same cortical location can be stimulated consistently (Bashir et al., 2011;Sack et al., 2009). For this purpose, individual anatomical magnetic resonance images (MRIs) were not required, as instead we relied on “hotspotting” to locate the FDI representation in M1. Where MEP protocols can range from 20 to 100 TMS pulses (Ahn & Fröhlich, 2021;Nitsche & Paulus, 2000;Schapira et al., 2022), we opted for 60 pulses as suggested for reliability in TEP measurements (Hernandez-Pavon et al., 2023). The total number of TMS trials falls within historical safety guidelines (Rossi et al., 2009) and the amount used for motor mapping (Weise et al., 2023). The combination of TMS and tDCS was expected to be safe (Rossi et al., 2021), although we are currently not aware of any prior reports on TMS with tDCS intensities used in this study.

#### 2.3.3 TEP measurement

EEG was recorded simultaneously with TMS-MEP measurements using a TMS-compatible amplifier (BrainVision, Gilching, Germany) and TMS-compatible Ag/AgCl sintered “C” electrodes (BrainVision, Gilching, Germany) attached to a custom EEG cap (EasyCap, Wörthsee, Germany) with cutouts for HD1 Electrode Holders (Soterix Medical, Woodbridge, NJ). A custom 32-channel electrode layout was used, with electrode positions redistributed around the HD1 cutouts (Fig. 2b). Because we were interested in TEPs at M1 (approximately C3 and C4), the adjacent positions were more densely populated. Electrode wires were wired away from C3 and C4 and oriented such that there was a 90 degree angle with the TMS coil in order to limit TMS artifacts in the signal (Sekiguchi et al., 2011). The EEG layout was symmetrical across the midline such that the recordings would not be biased toward either hemisphere. Data were sampled at 5 kHz and epoched around triggers sent by the TMS.

#### 2.3.4 tDCS

tDCS was delivered using the same layout used in our previous study (Hsu et al., 2023) (Fig. 2b). Using a high-definition (HD) tDCS system (M×N-9, Soterix Medical, Woodbridge, NJ), current was spread out across four electrode pairs to limit skin sensation, and electrodes were placed in a montage that optimizes electric field intensity at the M1 representation of the left-hand fingers. Ag/AgCl sintered ring HD electrodes (Soterix Medical, Wooodbridge, NJ) were attached to the head through the custom EEG cap with low-profile HD-tDCS electrode holders (Motamedi et al., 2023) to allow for proximity of the TMS coil to the scalp. Conductive gel (Signagel, Parker Laboratories, Fairfield, NJ) was applied on the scalp under the electrodes in the same manner as EEG preparation. Anodal stimulation (inward current on the targeted cortical structure) was delivered in the posterior-anterior direction with anodes placed over the right parietal lobe at P4, CP4, CP2, and P2, and cathodes placed over the center-right frontal lobe at F4, F2, AF4, and Fz. A reference electrode was placed over CP3. The current through each electrode pair was either 1 mA for a total of 4 mA, 1.5 mA for 6 mA total, or 0.25 mA for 1 mA total for sham stimulation. When the stimulator was activated, current intensity ramped up gradually to full intensity over 30 s, and similarly ramped down over 30 s when the stimulator was turned off. The tDCS device constantly monitored impedance during stimulation to ensure that every channel was below 10 kΩ, thus reducing the likelihood of an adverse reaction on the scalp. Once the current reached full intensity, we asked the subjects whether the sensation is acceptable, and if so, whether they would like to proceed to the task. Throughout the experiment, the participants were reminded that they can ask to end the stimulation at any point, for both TMS and tDCS.

The 0 mA group received 30-s ramped sham stimulation up to 1 mA total at the beginning, immediately followed by a 30-s ramp down. This low intensity was used because tDCS at 4 mA (and above) is very noticeable and cannot be reasonably sham controlled (Hsu et al., 2023), whereas the difference is more subtle at 1 mA. After stimulation had ended, the subjects were asked whether they thought they received verum or sham stimulation (or did not know). We did not expect to achieve comparable levels of placebo effects from skin sensation alone, since participants under the sham condition would experience significantly lower levels. Nonetheless, as discussed below in the Pilot Data section, we did not expect a correlation between sensation and motor performance. Sham stimulation at 1 mA may not be effective in a within-subject design (Learmonth et al., 2019;O’Connell et al., 2012;Turi et al., 2019;Wallace et al., 2016), but in this case, the subjects would not have multiple stimulation conditions to compare across. Therefore, we expected all subjects to perceive some level of stimulation.

#### 2.3.5 Motor sequence learning task

The subject performed the same task used in our previous study, following the same procedures and sequences (Hsu et al., 2023) (Fig. 2a, c). They were seated facing a computer monitor, with their left-hand fingers resting on a four-key response pad labeled with the digits “1”, “2”, “3”, and “4” from left to right. The monitor displayed a MATLAB-based graphical user interface (GUI). To avoid any possible experimenter bias, all instructions for the task were delivered through the GUI. During each trial of the task, the GUI showed a five-element sequence consisting of the digits “1”, “2”, “3”, and “4”, for example, “4-1-3-2-4” (Bönstrup et al., 2019;Karni et al., 1995). The subject was instructed to press the matching keys sequentially in the order shown on the GUI, as quickly and as accurately as possible. Each time any key was pressed, the GUI displayed an asterisk above the most recent digit in the sequence, regardless of correctness. The initial task was performed with the left (nondominant) hand using a first sequence (L:S1) and paired with tDCS. It was repeated 60 min later without stimulation to test for lasting effects. To test for any lasting effect on the unstimulated hemisphere, the task was then repeated without stimulation on the right hand on a new sequence (R:S2). We also tested a new sequence on the left hand (L:S3) to see whether tDCS can boost subsequent learning on the stimulated hemisphere.

#### 2.3.6 Sensation rating

After the stimulation was turned off, the subject was asked to rate skin sensation levels of tDCS perceived at three different time points of stimulation: at the beginning of stimulation, at the middle of stimulation, and after the stimulation is turned off. Ratings were recorded on a Wong–Baker visual analog scale (D. L. Wong & Baker, 1988) from 0 to 10, with 10 being the most severe. One or more qualities of the sensation were also rated from the options: “No sensation”, “Tingling”, “Pricking/Stinging”, “Itching”, “Burning”, “Other”. Subjects were asked whether they believed they received stimulation: “Do you think you received stimulation? (Yes, No, Not sure)”

The IRB protocol for TMS and tDCS included an adverse event reporting form which asked the participant to report any of the following: headache, neck pain, scalp pain, tingling, burning sensation, skin redness, sleepiness, trouble concentrating, acute mood changes, and nausea/lightheadedness/dizziness. These ratings are reported as secondary safety outcomes in this study (Supplementary Fig. S7).

#### 2.3.7 Data exclusion

Only data from subjects who have completed all TMS, MEP, EEG, and tDCS procedures were included. MEP and TEP analyses excluded trials that were three quartiles away from the median (in their log of power). MEP ratios were not calculated for recordings where fewer than 30 MEPs were detected. Outliers in task performance were also excluded. We excluded subjects who showed signs of inattentiveness, that is, overall accuracy less than 50% in keypresses, any consistent pauses longer than 3 s when keypresses were expected, or any trials where no response was recorded.

### 2.4 Analysis plan

Data were analyzed using MATLAB, following the plans detailed inTable 1andFigure 2. Power analyses were based on our previous behavioral data and described in more detail in the Pilot Data section. These previous data and pilot data were not included in the current study’s data set for analysis. The main performance outcome for each subject was calculated exactly as done previously (Hsu et al., 2023), by taking the average number of correct sequences (NCS) (i.e., 5 consecutive keypresses with no errors) completed by the subject during each 10-s trial, across all trials of the first iteration of the task concurrent with stimulation. MEP amplitudes for each subject were calculated by taking the median of the raw amplitudes across 60 EMG epochs triggered by the TMS pulses. The median only includes trials with a biphasic MEP detected using the MATLAB findpeaks() function. Inclusion criteria consist of a positive peak with a minimum Peak Prominence and maximum Peak Width, followed by a negative valley with a minimum Peak Prominence and minimum Peak Width. The peak had to occur within a time window after the TMS trigger (approximately 20–50 ms; lower latency is possible but not observed here). Specific criteria for peak identification were tested and refined while blinded to the stimulation conditions and finalized before unblinding for the final statistical analyses. Change in MEP amplitudes was calculated by taking the ratio of the post-tDCS median across trials over the pre-tDCS median across trials. These primary outcomes test hypotheses H1 on performance and H2 on change in MEP, and H3 tests for a correlation between these two effects.

**Table 1.** Planned analyses.

Question	Hypothesis	Outcome measures	Sampling plan	Analysis plan	Rationale for deciding the sensitivity of the test	Interpretation given different outcomes	Theory falsifiable by the outcomes
Does dose of concurrent tDCS have an effect on motor sequence learning?	H1: Performance differs with tDCS dose.	Average number of correct sequences (NCS) with left hand on sequence S1 (L:S1)	N = 40 per group yields >90% power	Linear model with intensity as a graded variable and typing speed as covariate. If no significant effect is found: see Figure 3 .	Effect size of Cohen’s *d* = 0.56 between 4 mA and 0 mA from previous data ( Hsu et al., 2023 ) was used as the basis for power analysis.	*p* > 0.05: see Figure 3 for follow-up analyses. *p* < 0.05: tDCS effect on behavior is monotonic with intensity.	tDCS modulates learning-related plasticity.
Does dose of concurrent tDCS have an effect on corticospinal excitability?	H2: Monotonic increase of MEP change with tDCS dose.	Post–Pre MEP amplitude ratio	Assuming *η* ^2^ = 0.12, power = 95%	Linear model with intensity as a graded variable.	Effect size assumed here is much more conservative than a previous study ( *η* ^2^ = 0.91; Ahn and Fröhlich, 2021 )	*p* > 0.05: nonlinear or no effect on MEP *p* < 0.05: monotonic effect on MEP	tDCS modulates cortical excitability.
Is motor sequence learning associated with changes in MEP?	H3: Performance correlates with MEP change.	NCS, post–pre MEP amplitude ratio	N = 40 points per group can resolve a significant association with 90% power and *r* ≥ 0.29	Linear mixed effects model with MEP as fixed effect and subject as random effect	Assuming a fixed N as computed above, we determined resolvable effect size.	*p* < 0.05 is consistent with a linear effect of tDCS on a common cause of MEP change and motor learning.	Effects of tDCS on MEP and motor skill are similar and share the same neural substrate.

See Pilot Data section for details.

**Fig. 3.**
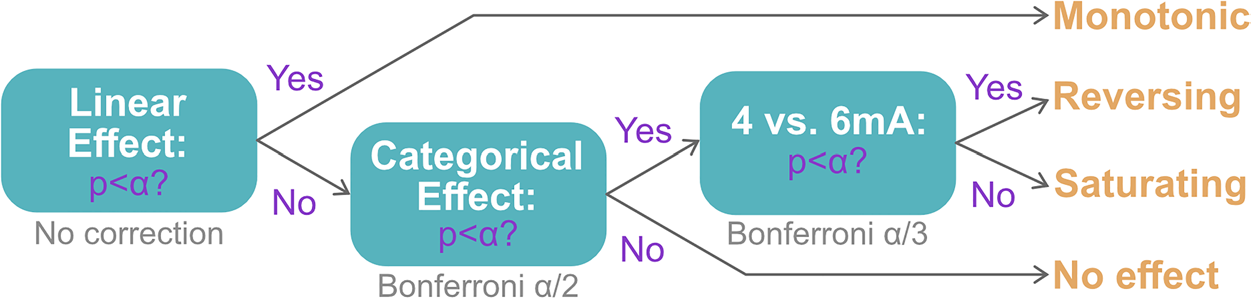
Analysis pipeline for primary behavioral outcome. An initial linear model at the first stage of analysis uses intensity as a graded variable and typing speed as a covariate. If no significant effect was found, a second stage analysis would use intensity as a categorical effect. If a significant effect was found, a third stage analysis would apply a Tukey HSD pairwise comparison between 4 and 6 mA. Second and third stage analyses would be Bonferroni-corrected accordingly, with α = 0.05. The interpretations of the dose effect given these possible outcomes are “monotonic”, “reversing”, “saturating”, and “no effect”.

Because we have prior data on behavior, we applied a more rigorous analysis pipeline for this outcome (Fig. 3). If a significant effect was found in the initial linear model with intensity as a graded variable (and typing speed as a covariate), intensity would be interpreted as a monotonic effect, since we did not expect to resolve a significant difference between 4 and 6 mA. In the case where no significant effect was found at the first stage, we would test a follow-up linear model with intensity as a categorical variable. A significant finding at this second stage would be followed by a Tukey HSD pairwise comparison between 4 and 6 mA. A significant difference between the two groups would be interpreted as a “reversing” effect, whereas a lack of a difference would be interpreted as a “saturating” effect, with linearity ruled out. A nonsignificant finding at the second stage would be interpreted as a lack of effect from tDCS intensity. Post hoc analyses in the second and third stages would be Bonferroni-corrected accordingly. In the case of a null finding resulting in a saturating dose–response or no effect, we would use Bayes factor analysis to measure the evidence in support of the corresponding null hypothesis using an established MATLAB toolbox (Krekelberg, 2019).

Exploratory analyses included a test for lasting effects and carryover effects as observed in our previous study (Hsu et al., 2023). In that study, we did not find a significant performance difference after 1 h nor between the different hands or sequences. We tested whether these effects replicated in the current study by repeating the same linear model on the number of correct sequences. MEP and TEP effects on the right hand were exploratory. Although they may serve as a within-subject control, there may have been behavioral carryover effects due to intermanual transfer (Dickins et al., 2015), and the high tDCS intensities may have caused parts of the left hemisphere to be stimulated. We implemented mixed-effects models that considered tDCS intensity, left/right hemisphere, pre-/post-tDCS as main factors, and potential interactions between these, as well as a random effect of subjects. We had no specific hypothesis, but we at least observed whether there were any changes in MEP and TEP amplitudes on the right hand. We were interested in whether there was an effect of tDCS dose on changes in TEP, and whether those effects were hemisphere specific. We also sought to observe any possible correlation between performance, MEP, and TEP effects. In general, we looked at post–pre local mean field power (LMFP) around the region of interest around C4 and C3, sampled from the electrodes in the vicinity (Fig. 2b), as well as global mean field power across all channels. As an a priori measure, we expected to see a significant across-group difference in post–pre LMFP ratio within the 25–60 ms period after the TMS pulse, as reported by Ahn and Frohlich (2021). EEG data were processed using original scripts and the EEGLAB package for MATLAB (Delorme & Makeig, 2004). All results were evaluated at a significance level of α = 0.05. If any significant dose effect was found, post hoc analysis consisted of Tukey HSD to evaluate pairwise differences between individual groups. We report descriptive statistics for samples organized by time of day (morning vs. afternoon), age, sex, and stimulation condition.

### 2.5 TEP analysis

We did not commit to any particular analysis pipeline for TEP data, as this was an exploratory method that we were not experienced in. Analyses primarily used functions from the TMS-EEG signal analyzer (TESA) toolbox for EEGLAB (Mutanen et al., 2020;Rogasch et al., 2017). EEG recordings were epoched to 0.5 s before and after the onset of the TMS pulse, which we defined as*t*= 0.

Due to the large volume, individual epochs were not inspected manually. Prestimulation epochs were combined with poststimulation epochs for processing to ensure consistency. TESA functions were applied to remove the TMS artifact from -3 to 10 ms, followed by subtraction of the baseline between -500 and -400 ms. The EEGLAB FastICA algorithm was used to sort the epochs into independent components, which were automatically classified by TESA. Any components identified as TMS-evoked muscle activity, eye blinks, eye movements, muscle activity, electrode noise, and sensory artifacts were automatically removed without manual inspection. We also filtered out components with a large step function. To control against outlier trials and high variability, the median TEPs were taken across trials following ICA removal of artifacts.

C2 and C4 were selected as ROI channels because they are located above the right M1 and maintained consistent polarity throughout our recordings (Fig. 14), presumably capturing activity related to the left hand. Analyses were performed on peaks of interest, around 30, 45, 60, 100, and 180 ms after the TMS pulse, that are thought to represent genuine neural responses (Hernandez-Pavon et al., 2023). Here we picked the specific times that most closely match peaks in the current data (Fig. 9a;Supplementary Fig. S9a): 33, 45, 55, 100, and 175 ms post-TMS.

Averaging over a 10-ms window around the peak times (dashed lines inFig. 15a) and across C2 and C4, we compared the difference between post- and prestimulation TEPs across groups. Based on Lilliefors tests within groups, we determined that TEP amplitudes were not normally distributed. Thus, for pre- and poststimulation comparisons, we applied Wilcoxon signed rank tests, and for across-group comparisons, we applied Kruskal–Wallis tests (Fig. 15). Comparisons across pre- and poststimulation TEPs were done across all subjects in order to identify channels where significant changes occurred.

### 2.6 Deviations from protocol

For the analysis of H2, we had originally planned to take the mean MEP amplitude across trials, but we instead chose to take the median across trials. We believe this choice was justified because we found through Lilliefors tests that most subjects had at least one set of MEP amplitudes that were not normally distributed across trials (within the same time point on the same hand). Furthermore, taking the mean or median does not affect the final outcome for H2.

The same experimenter who operated the tDCS stimulator also prepared the participant for tDCS, specifically by connecting the stimulation electrodes to the cap and to the stimulator. However, this experimenter did not otherwise interact with the participant. This change was made to minimize errors in polarity settings and connections that may be caused by miscommunication between experimenters. To build in redundancy in the blinding procedure, that experimenter also kept records of the electrode connections and stimulator settings, including photographs of the stimulator display. For simplicity of the equipment setup, triggers were not sent from the TMS to the EEG amplifier. Based on pilot testing, we determined that the TESA toolbox for EEGLAB (Mutanen et al., 2020;Rogasch et al., 2017) reliably detects the onset of a TMS pulse with a precision of within 1 ms from the recorded trigger time, making the direct trigger redundant. Although we did not originally specify the EMG sampling rate in our preregistration, we had intended to sample at 2 kHz. However, due to a hardware failure in the neuronavigation and EMG equipment after we completed data collection for Subject 5, the recording settings had to be reconfigured on a new computer. We failed to notice that the EMG had been recorded at 500 Hz by default and changed it to 2 kHz starting with Subject 57. In our hands, TMS artifacts in TEP can vary considerably across subjects and electrodes. For the exploratory TEP analyses, we originally intended to exclude electrodes that showed stimulation artifacts lasting more than 25 ms. Because TEP-evoked muscle activity can be hard to distinguish from the TMS artifact and as mentioned above, we had a large volume of data, we did not manually or automatically filter out EEG channels and epochs.

## 3 Pilot Data

Pilot data were collected prior to the preregistered study and not included in the current analyses.

### 3.1 tDCS at 6 mA

**Fig. 4.**
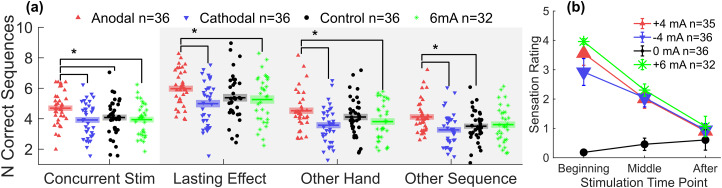
Preliminary results combining published results with a 6 mA group that was collected later. (a) Overall performance in the 6 mA group was not significantly different from those of the -4 and 0 mA groups. Each point represents performance by one subject averaged across all trials. Horizontal bars represent group average and shaded areas represent standard error of the mean (SEM). Brackets and asterisks represent differences with*p*< 0.05. (b) Sensation ratings rated on a visual analog scale from 0 to 10, with 10 being the most severe. Average sensation ratings for 6 mA were slightly higher, but not significantly different from +4 to -4 mA ratings. Error bars represent SEM.

Following our findings of a positive effect on skill learning performance at 4 mA (Fig. 4a) (Hsu et al., 2023), we collected an additional group of N = 32 that received 6 mA tDCS using the same protocol, under approval by the City University of New York Institutional Review Board. Current flow modeling using ROAST (realistic volumetric approach to simulate transcranial electric stimulation;Huang et al., 2019) was conducted a priori in consideration of safety. Simulations were run on the same 10 anatomical MRIs we used in our previous study to formulate the electrode montage (Hsu et al., 2023). Applying the 1.5 mA per electrode pair under our previous configuration for a total of 6 mA, we found that the maximal electric field achieved on the surface of the brain was approximately 1.8 V/m, which corresponds to an estimated current density of 0.23–0.50 A/m^2^in the brain (Huang et al., 2013). This is well below the threshold for tissue damage at approximately 50 A/m^2^for 30 min (Bikson et al., 2016); in other words, >100 mA would be necessary to induce hazardous levels of current density in the brain. While currents as low as 4 mA passed through a single electrode may cause adverse effects (Chhatbar et al., 2017;Khadka et al., 2020;Workman et al., 2020), here we limited stimulation to 1.5 mA per electrode. Additionally, the base area of the gel in the Soterix HD1 Holder is approximately 4.5 cm^2^, through which a current of 1.5 mA would yield approximately 0.33 mA/cm^2^current density on the skin per electrode. Since this is below the upper tolerability limit of 0.5 mA/cm^2^commonly cited in the iontophoresis literature (Prausnitz, 1996), we expect this level of stimulation to be tolerable.

A one-way ANOVA on the behavioral outcomes showed a significant effect of current on performance (mean number of correct sequences per trial) during the initial training task (*F*(3,136) = 3.76,*p*= 0.012). We found in a post hoc Tukey’s HSD test that the 6 mA group performance was not significantly different from those of the -4 and 0 mA groups (*p*= 1.0,*p*= 0.96, respectively), resulting in a nonmonotonic relationship overall (Fig. 4a). Contrary to our hypothesis, 6 mA did not appear to confer any benefit over no stimulation. However, we suspect that there may have been inhomogeneity across the different cohorts, since the data were collected over 1 year apart on a new cohort of subjects. Replication of the previous experiment and validation of these results were, therefore, an important goal of the planned experiment. The 6 mA tDCS was well tolerated at a rating of around 4 on a visual analog scale from 0 to 10 (D. L. Wong & Baker, 1988) (Fig. 4b), with no difference in sensation rating at the beginning of stimulation from -4 to +4 mA (one-way ANOVA;*F*(2,100) = 1.59,*p*= 0.21).

### 3.2 Power analysis and predicted outcomes

#### 3.2.1 Hypothesis H1: Performance differs with tDCS dose

**Fig. 5.**
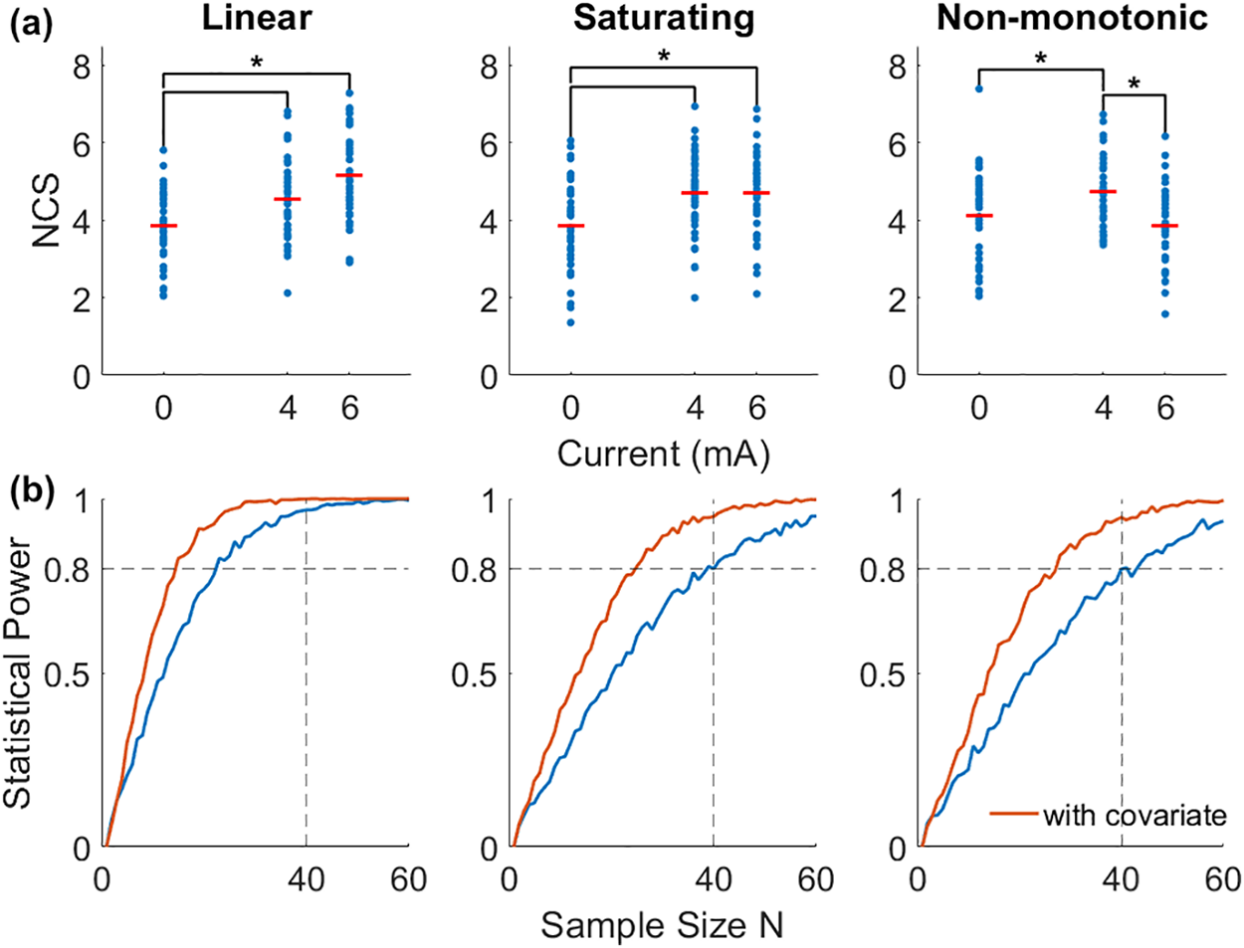
Simulation of possible behavioral dose–responses. (a) An example of simulated results with N = 40 in each group. Samples are jointly distributed based on the covariance between NCS from the preliminary data and typing speed from the online experiment. Mean values for 6 mA in the first scenario were linearly extrapolated from the preliminary data. Mean values for the second scenario were set with equal values for 4 and 6 mA. Mean values for the third scenario were set equal to those from the preliminary data. Brackets and asterisks indicate significant differences (α = 0.05) found in post hoc Tukey HSD. (b) Estimated statistical power of yielding*p*< 0.05 from an*F*-test on the linear model without (blue) and with (red) the typing speed covariate, across 1,000 simulations, calculated for sample sizes from 1 to 60. The desired threshold was set at 80% power.

Based on the observed positive effect of 4 mA tDCS, we predicted three possible outcome scenarios in the planned experiment with regard to hypothesis H1: (1) that there is a linear relationship between performance (measured as mean NCS throughout the initial task) and current intensity; (2) that there is a saturating but monotonic relationship, where the outcomes of 6 mA and 4 mA are not significantly different, but higher than that of 0 mA; or (3) that there is in fact a nonmonotonic relationship as observed in the preliminary data, where the increase in intensity from 4 mA to 6 mA has a reversing effect. In order to detect any of these dose effects, we applied a linear model with the current intensity group as a graded fixed effect variable. We found through an online experiment (see below: “Predicting motor learning ability”) that a subject’s typing speed is positively correlated with the mean NCS throughout the learning task (*r*= 0.62). Therefore, our linear model adjusted for typing speed as a contributing factor, which we expected to improve statistical power. We powered the experiment at 80% by determining sample size through simulations of each of the three predicted outcome scenarios. A total of 1,000 randomized iterations were run in MATLAB for each scenario, repeated over increasing sample sizes from 1 to 60 (Fig. 5a). Using the Lilliefors test on the prior data, we determined that the mean number of correct sequences during the initial task was normally distributed in the 0, 4, and 6 mA groups. The typing speeds collected from the online experiment were likewise normally distributed. Thus, simulation data were randomly drawn from a joint normal distribution using the covariance between NCS from the preliminary data and typing speeds from our online experiment. These data assume the previous effect size of Cohen’s*d*= 0.56 between the 0 and 4 mA conditions (Hsu et al., 2023). For the linear case, we extrapolated the mean value to the 6 mA group (*d*= 0.92). For the saturating case, we used the same mean values for 4 mA and 6 mA, and for the nonmonotonic case, we use the same exact values from the preliminary data (Fig. 4a, concurrent stim.). A “successful” outcome was defined as one with a*p*-value less than α = 0.05 from an*F*-test on the model, indicating a significant effect of tDCS dosage. The statistical power for each sample size was thus determined by taking the percentage of successful simulations (Fig. 5b). At N = 40, even without the typing speed covariate, the statistical power of our model is close to 80% in all three predicted scenarios. When adjusted for typing speed, the statistical power is over 90% in all three scenarios.

#### 3.2.2 Hypothesis H2: Monotonic increase of MEP change with tDCS dose

We tested for an effect on excitability by fitting a linear model on the post-/pre-MEP ratio with tDCS intensity as a graded fixed effect. Using G*Power (Faul et al., 2009), we determined that with N = 40, even a modest effect of tDCS on MEP change with partial*η*^2^= 0.12 (relative to*η*^2^= 0.91 found byAhn & Fröhlich, 2021) is sufficient to yield 95% power for hypothesis H2 (α = 0.05). A significant finding for H2 would indicate a linear effect of tDCS intensity on MEP, whereas a null finding would suggest a nonlinear effect or no effect on MEP.

#### 3.2.3 Hypothesis H3: Performance correlates with change in MEP

**Fig. 6.**
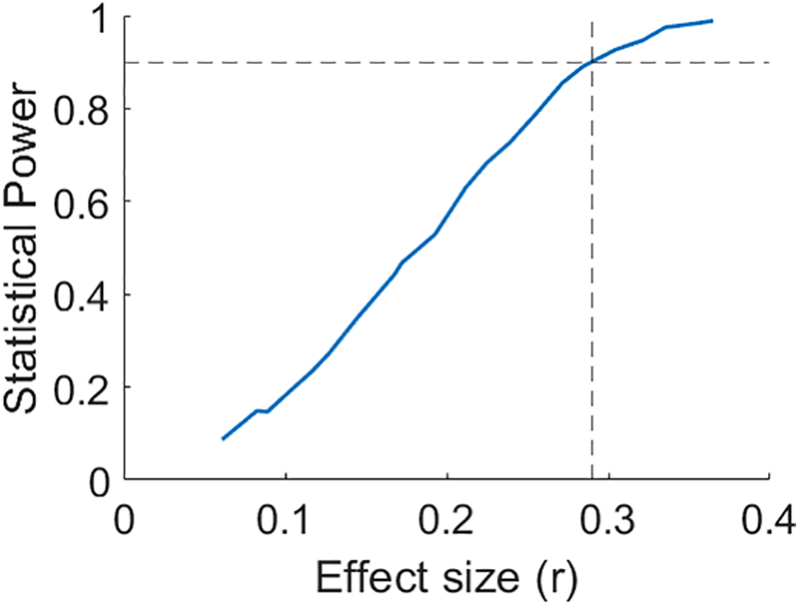
Simulation of a common neural substrate with linear effects on both performance and MEP. N = 40 samples per group of MEP and performance measurements were randomly generated based on the model presented inFigure 1. We assumed a linear effect*a*of electric field intensity on a neural substrate that in turn has equal linear effects*b1*=*b2*=*b*on the outcome measures. Since the Pearson correlation coefficient r between MEP and performance is proportional to*b*, we use r as a measure of effect size. Across 1,000 simulations, we find that we can detect a significant association with 90% power when there is a correlation of at least r = 0.29.

As power analysis for hypothesis H3, we simulated N = 40 samples per group following the causal model presented inFigure 1. We assumed a linear effect of tDCS-induced electric field onto the common learning-related neural substrate (arrow into white box inFig. 1, denoted here as “*a*”). This common cause in turn linearly affects MEP and performance (denoted “*b1*” and “*b2*” here; we set*b1*=*b2*=*b*in the simulation). We added normally distributed observation noise (with unit standard deviation) to both outcome variables, plus a random offset per subject. We fitted a linear mixed effects model for performance with MEP as a fixed effect and subjects as a random effect, then repeated the simulation 1,000 times to estimate power, that is, the likelihood of obtaining a significant effect. Increasing the strength of*b*increases the correlation between performance and MEP. Thus, the Pearson correlation coefficient serves as a measure of effect size. With a significance threshold of α = 0.05, we expect to observe a significant association between performance and MEP with 90% power at approximately r = 0.29 (Fig. 6). This estimate is valid for linear effects (*a*,*b1*,*b2*, with*b1*=*b2*) with no direct effects of tDCS on MEP or direct effects between MEP and performance. However, a significant association is also possible for nonlinear common-cause effects (*b1*,*b2*) provided they are collinear (i.e., the nonlinear dependence on stimulation intensity has the same form). A null finding for H3 may rule out a linear/collinear common-cause effect, but it is also possible that direct effects cancel a common cause. Finally, a positive result is also possible due to electrical fields effects via separate mechanisms (direct arrow from field to MEP inFig. 1). We did not expect H3 to hold if H1 and H2 showed no effects. In short, a positive finding for H3 is consistent with, but does not prove a common physiological substrate for H1 and H2. A null finding for H3 would make it more difficult to argue that there is a common cause, but does not rule it out. Only direct observation of the neural substrate can answer this question.

### 3.3 Sensation confound

We were also concerned that a potential tDCS dose–response may be confounded by a sensation effect. Without matched controls, a positive correlation between physiological or behavioral outcomes and skin sensation could suggest that the tDCS effect is at least partly driven by a sensation placebo effect. Conversely, a negative correlation could suggest that stronger sensations caused by higher intensity stimulation may have a detrimental effect when the subject is more distracted during training. From our preliminary data, we observed slightly higher skin sensation levels in the 6 mA group than in the +4 and -4 mA groups (Fig. 4b). We fitted a linear mixed effects model over the initial task performance (number of correct sequences) with a fixed effect of sensation and random intercept of groups, excluding the 0 mA group. The estimate of the sensation fixed effect was*β*= -0.063, with*p*= 0.17 and 95% confidence interval between -0.15 and 0.026, suggesting no effect of sensation on performance. We repeated this analysis on the final outcome to test whether there were any nonlinear effects due to variation in attention.

### 3.4 Predicting motor learning ability

**Fig. 7.**
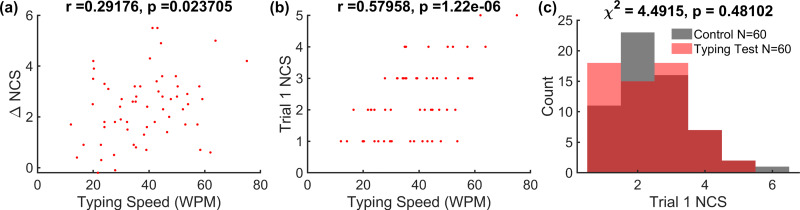
Typing speed as a predictor of baseline motor skill and skill learning ability. The same motor skill learning task was performed online on two cohorts of N = 60 subjects, one with and the other without a preceding typing test. (a) Pearson correlation of performance gain (from first trial to last 10 trials) with typing speed. (b) Pearson correlation of baseline skill performance during the first trial with typing speed. (c) Comparison of distributions (chi-squared test) of baseline skill performance in subjects who completed a baseline typing task beforehand and those who did not.

It is possible that some participants have generally better dexterous motor skills, resulting in better performance at the outset of the sequence training and/or quicker improvement during training. Therefore, as an additional control for homogeneity in motor skill between groups, we measured performance in a baseline task preceding the main trial (seeFig. 2c). We used the typing test described above in Methods. This task was followed by the same motor sequence learning task to be used in the main experiment, with the exact same sequence (4-1-3-2-4) that will be trained on when tDCS is applied simultaneously. Sixty right-handed adults were recruited to complete this pilot experiment, through an online human subject research platform (Prolific, London, UK). We found that the typing speed metric is positively correlated with learning gain (*r*(58) = 0.29,*p*= 0.024;Fig. 7a) as well as baseline performance (*r*(58) = 0.58,*p*< 0.001;Fig. 7b) in our motor sequence learning task. To test whether the typing test may have a priming effect on the motor learning task, we conducted an additional online experiment with 60 right-handed adults who only performed the motor sequence learning task. Using a chi-squared test, we found that there was no difference in distribution of baseline performance during the initial typing test across groups (*χ*^2^= 4.5,*p*= 0.48, N = 60 per group;Fig. 7c). Based on these results, we determined that a short and simple typing test suffices as a baseline test to predict motor sequence learning performance, while being different enough from the main task such that task performance is not affected.

## 4 Results

The experimental setup and methods are summarized inFigure 2and described in detail in the Methods section.

### 4.1 Planned analyses

**Fig. 8.**
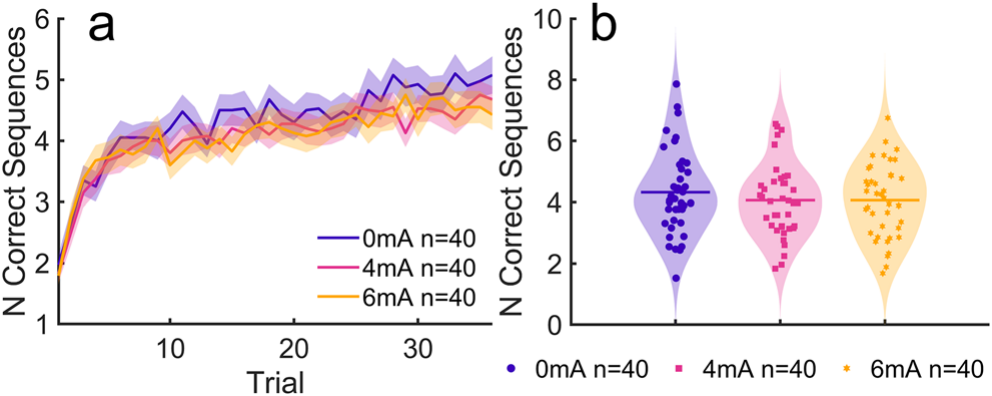
Motor performance under different stimulation conditions. (a) Motor performance was measured as the number of correct sequences (NCS) completed per trial, shown here for the initial learning task concurrent with tDCS. Performance typically improves rapidly in around the first 10 trials, after which it saturates over the rest of the task. Each trial lasted 10 s, spaced apart by a 10-s break (12 min total). (b) Average NCS throughout the initial task are represented here as points for each individual subject. Bars indicate the mean NCS within each group and shaded areas represent kernel density estimates.

**Fig. 9.**
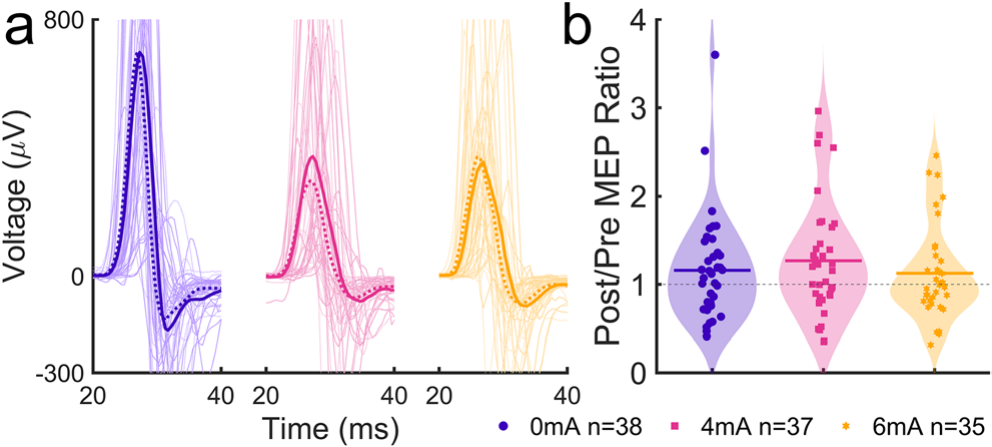
Change in MEP amplitude following different stimulation conditions. (a) Pre- and post-tDCS MEP recordings, epoched around time = 0 at the TMS trigger. Thin lines represent median MEPs across trials for individual subjects and bold lines represent mean MEPs across subjects within groups. Dotted lines represent prestimulation and solid lines represent poststimulation. (b) Post-/prestimulation MEP amplitude ratios. Points represent individual subjects, bars represent within-group means, and shaded areas represent kernel density estimates.

Planned statistical analyses for the primary hypotheses follow the Analysis Plan detailed below in the Methods section. Motor skill learning was assessed on the left hand for the initial task, while tDCS was applied to the right M1 (Fig. 2, L:S1 + tDCS in the timeline). Performance was measured as the number of correct sequences (NCS) averaged over all 36 trials (Fig. 8). This outcome measure captures speed, accuracy, and improvement throughout the task (Supplementary Fig. S1). Corticospinal excitability was measured as MEP amplitudes (Fig. 9a) and changes were assessed as the ratio post-tDCS over pre-tDCS (Fig. 9b).

#### 4.1.1 H1: No effect of tDCS dose on motor performance

With current intensity as a graded variable and typing speed as a covariate, a linear model fitted on the average NCS across trials reveals no effect of tDCS intensity (*F*(1,117) = 2.12,*p*= 0.148,Fig. 8b). Similarly, a subsequent linear model with intensity as a categorical effect also indicates a lack of an intensity effect (*F*(1,117) = 1.07,*p*= 0.347). A Bayes factor of*BF_01_*= 8.48 in favor of the null hypothesis provides moderate evidence that 4 and 6 mA did not modulate motor learning performance. In follow-up analyses, there was no significant difference in motor performance based on time of day of the experiment, participant sex, and participant age (Supplementary Fig. S2). Even when comparing performance gain between the first trial and the last 10 trials, there was no effect of tDCS dose (Supplementary Fig. S3).

#### 4.1.2 H2: No effect of tDCS dose on MEP

With current intensity as a graded variable, a linear model fitted on the post-/pre-MEP ratio indicates a lack of an intensity effect (*F*(1,108) < 0.001,*p*= 0.993,Fig. 9b). A Bayes factor of*BF_01_*= 13.4 in favor of the null hypothesis provides strong evidence that 4 mA and 6 mA did not modulate corticospinal excitability. We found that the change in MEP was not correlated with the total number of keypresses during the initial trial (Supplementary Fig. S4a), suggesting that it was not related to neuromuscular fatigue. A model intercept estimate of 1.19 (*t*(109) = 12.8,*p*< 0.001,*SEM*= 0.0541), that is, a post-/pre-MEP ratio following stimulation and training greater than 1, indicates an overall increase in MEP amplitude under all conditions. Likewise, the post-/pre-MEP ratio in the right hand was greater than 1 (Supplementary Fig. S5).

#### 4.1.3 H3: No correlation between motor performance and MEP

**Fig. 10.**
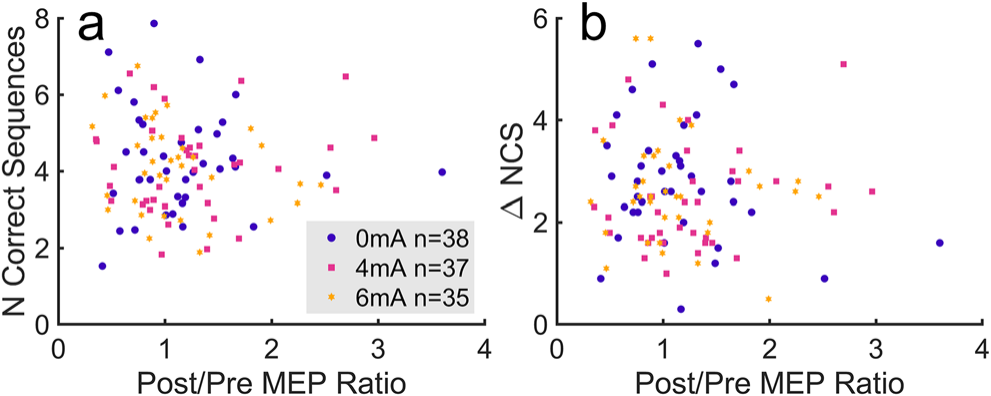
Relationship between motor performance and change in MEP amplitude. Individual points represent individual subjects. (a) Average NCS plotted against post-/prestimulation MEP amplitude ratio. (b) Difference in NCS between the last 10 trials and the first trial plotted against post-/prestimulation MEP amplitude ratio.

With post-/pre-MEP ratio as a fixed effect and subjects as a random effect, a linear mixed effects model indicates no significant effect of MEP on average NCS (*F*(1,108) = 0.118,*p*= 0.731,Fig. 10a). As expected, because H1 and H2 did not hold, H3 did not hold either. A Bayes factor of*BF_01_*= 22.9 for the model provides strong evidence that motor performance is not correlated with change in corticospinal excitability. Although there does not appear to be a linear/collinear common-cause effect between MEPs and motor performance, as stipulated in the analysis plan, we cannot completely rule out a nonlinear common-cause effect.

### 4.2 Exploratory analyses

#### 4.2.1 Alternative outcome measures for learning performance

Our planned outcome measure of behavioral performance was the number of correct sequences averaged over all 36 trials. Previous studies also measured performance difference between the beginning and end, or performance at the end of the sequence learning task, as a metric of performance gain. There was no significant difference in NCS in the mean over the last 10 trials across tDCS groups (Supplementary Fig. S6a;*F*(1,118) = 1.85,*p*= 0.177). We also found no significant effect of MEP ratio on NCS increase (Fig. 10b;*F*(1,108) = 0.438,*p*= 0.509,*BF_01_*= 19.8), Trial 1 NCS (Supplementary Fig. S6b;*r*(118) = -0.050,*p*= 0.60), or final NCS (average of last 10) (Supplementary Fig. S6c;*r*(118) = -0.081,*p*= 0.40).

#### 4.2.2 Counterbalancing and predicting baseline performance

**Fig. 11.**
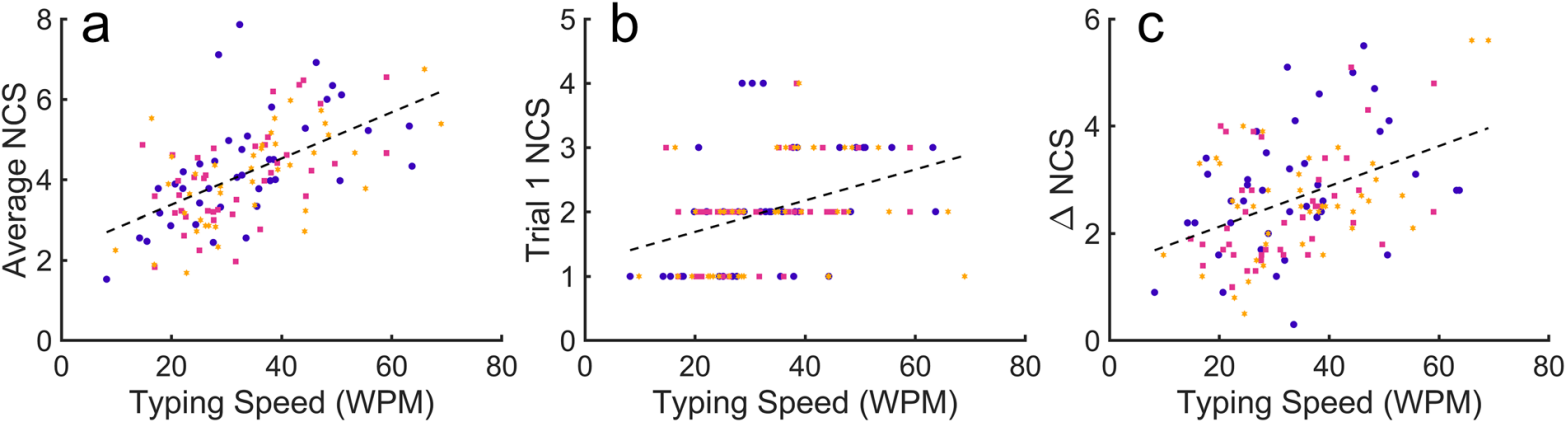
Relationships between typing speed and motor performance. Points represent individual subjects and dashed lines represent linear regressions across all samples. Bullet, square, and asterisk symbols correspond to 0, 4, and 6 mA groups, respectively. (a) Average NCS versus typing speed. (b) NCS during the first trial versus typing speed. (c) Change in NCS from the first trial to the last 10 trials versus typing speed.

A one-way ANOVA of typing speed across groups indicates that there was no difference in typing speed (*F*(1,118) = 0.127,*p*= 0.723). Furthermore, a one-way ANOVA of NCS in the first trial found no difference in baseline performance across groups (*F*(1,118) = 0.438,*p*= 0.510). Consistent with our pilot online experiments, typing speed positively correlated with multiple aspects of motor skill learning, including overall performance (*r*(118) = 0.569,*p*< 0.001,Fig. 11a), baseline performance (*r*(118) = 0.369,*p*< 0.001,Fig. 11b), and performance gain (*r*(118) = 0.421,*p*< 0.001,Fig. 11c). These results demonstrate that the typing test and group sorting algorithm were successful in predicting and counterbalancing baseline performance for homogeneous group assignments. The mean typing speed across all subjects was mean ± SEM = 33.5 ± 1.1 words per minute (WPM). Typing speed did not correlate with baseline MEP amplitude (Supplementary Fig. S4b).

#### 4.2.3 Sensation effects

**Fig. 12.**
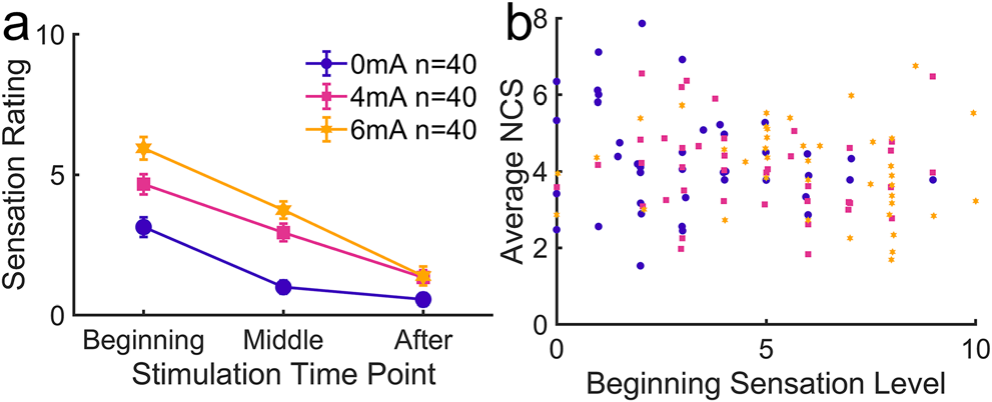
Sensation effects of tDCS. (a) Sensation intensity ratings at different time points on a visual analog scale from 0 to 10, with 10 being the most intense. “Beginning” refers to sensation after the initial 30-s ramp up, “Middle” refers to sensation around the middle of the 12-min stimulation period, and “After” refers to sensation after the stimulation was completely turned off at the end. Ratings were collected immediately after stimulation. Points represent within-group means and error bars represent standard errors of the means. (b) Average NCS of each subject plotted against the sensation intensity rating at the beginning of the stimulation. No significant correlation was found.

There were no dropouts during tDCS and the concurrent learning task. At the beginning of the stimulation, 4 and 6 mA were on average rated approximately 5 and 6, respectively, on a visual analog scale from 0 to 10, with 10 being the most severe (Fig. 12a). These values correspond to a “moderate” level. As expected, sensation level was rated considerably higher under the active stimulation conditions than under sham stimulation (with a 1 mA ramp at the start and end of stimulation). We fitted a linear mixed effects model on average NCS with sensation and current as continuous fixed effects and subjects as a random effect. There was no significant effect of sensation (*F*(1,116) = 2.03,*p*= 0.157) and no interaction between sensation and current intensity effects (*F*(1,116) = 0.434,*p*= 0.511). A Bayes factor of*BF_01_*= 5.12 for the sensation effect provides moderate evidence in favor of the null hypothesis. There was no significant correlation between performance and sensation ratings at the beginning (Fig. 12b,*r*(118) = -0.164,*p*= 0.0730). Various ratings were obtained to characterize the quality of sensations (Supplementary Fig. S7). The sensation that related to tDCS stimulation intensity was primarily “tingling” and “burning sensation”.

#### 4.2.4 Carryover effects on subsequent learning

**Fig. 13.**
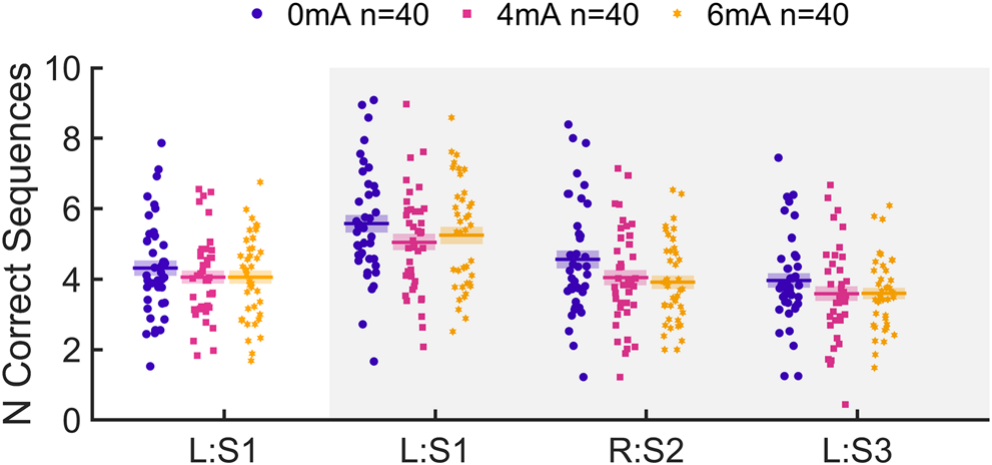
Motor performance in all learning tasks. Points represent individual subjects, horizontal bars represent within-group means, and colored shaded areas represent SEM. The initial learning task was performed concurrently with tDCS, using the left hand and training on sequence S1 (L:S1). The data shown in the unshaded area are the same as those inFigure 8. The shaded area denotes follow-up tasks performed 1 h after the end of the initial task and tDCS, in the order shown here from left to right. First, the same trained sequence S1 was repeated on the left hand, followed by a new sequence S2 on the right hand, and finally a new sequence S3 was trained on the left hand.

Through linear models similar to that applied for H1, we find no difference in motor performance across groups in the follow-up tasks using the left hand (Fig. 13) training on the same trained sequence (L:S1,*F*(1,117) = 3.17,*p*= 0.0777,*BF_01_*= 7.05) or on an untrained sequence (L:S3,*F*(1,117) = 3.68,*p*= 0.0574,*BF_01_*= 4.66). There was a significant effect of tDCS current intensity on motor performance in the follow-up task using the right hand, training on a new sequence (R:S2,*F*(1,117) = 8.28,*p*< 0.001), but a Bayes factor of*BF_01_*= 1.45 slightly favors the null hypothesis and warrants caution in interpreting this as a real effect. Motor performance measured alternatively as finger tapping speed also sees no effect of tDCS dose in any of the tasks (Supplementary Fig. S8).

#### 4.2.5 Effects on TEP

**Fig. 14.**
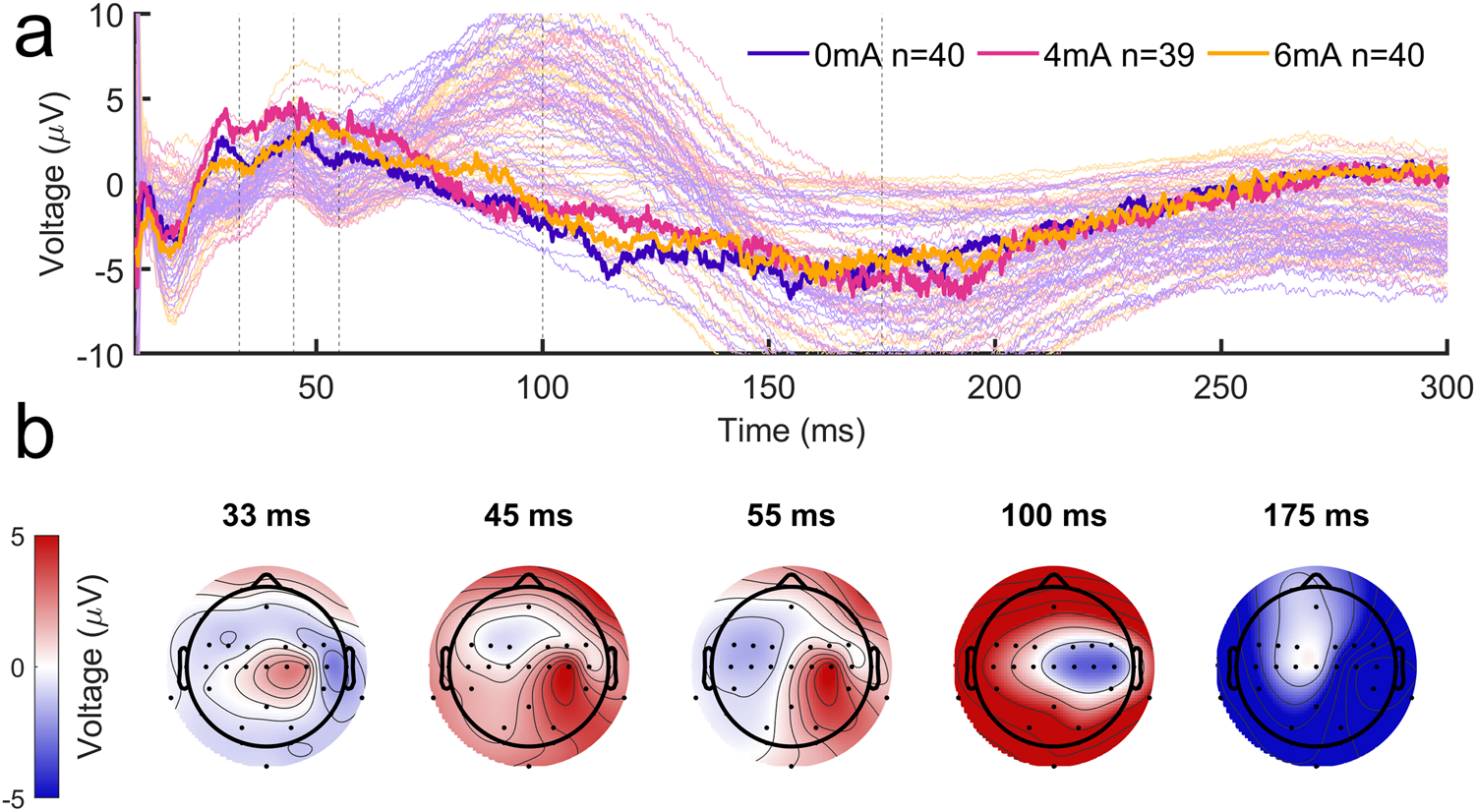
Time course of TEPs of right M1 and left hand prior to tDCS and training. (a) Thin lines represent prestimulation TEPs from individual channels, averaged across subjects within each group. Bold lines represent median prestimulation TEPs across subjects within each group, averaged across right M1 channels C2 and C4. (b) Topographical representations of TEP peaks corresponding to conventionally reported TEP components from the literature (P30, N45, P60, N100, and P180).

The TEPs in response to TMS over both hemispheres exhibit mirrored spatial distributions (Fig. 14b;Supplementary Fig. S9b) and similar TEP peaks at 33, 45, 55, 100, and 175 ms. (Fig. 9a;Supplementary Fig. S9a). Because we are primarily interested in the tDCS stimulated hand, we focused on a region of interest (ROI) over right M1 (channels C2 and C4).

**Fig. 15.**
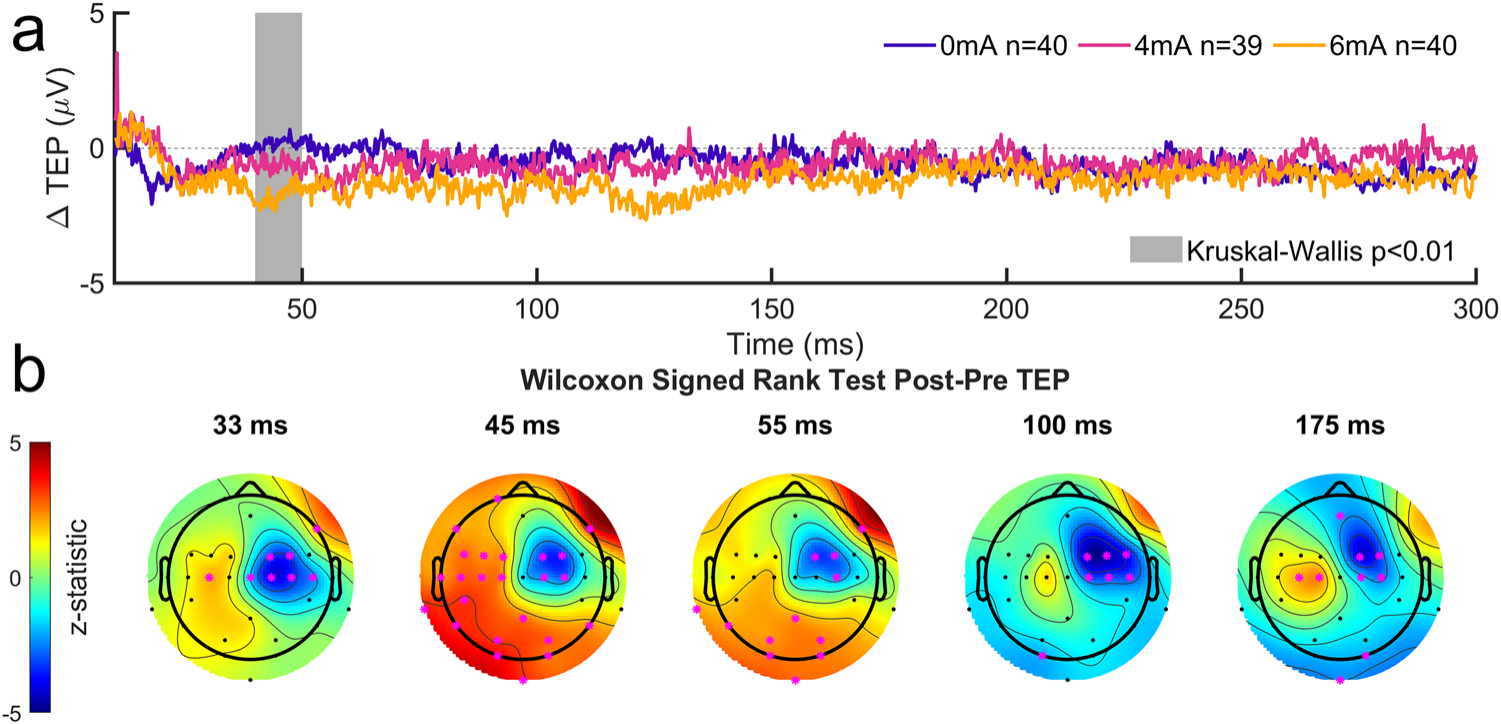
Post–Pre changes in TEPs following stimulation of right M1 and left hand. (a) Median post–pre difference in TEPs across subjects within each group, averaged across right M1 channels C2 and C4. Shaded areas represent time points where TEPs averaged over a 10-ms window around the time point were significantly different across groups (*p*< 0.01, Bonferroni corrected) in a Kruskal–Wallis test. (b) Topographical representations of z-statistics from Wilcoxon signed rank tests comparing pre- and poststimulation TEP amplitudes averaged across all subjects and over 10-ms windows around peak times corresponding to conventionally reported TEP components from the literature (P30, N45, P60, N100, and P180). Pink asterisks represent channels where a significant post–pre difference was found (*p*< 0.05).

Significant post–pre changes were found in the right M1 ROI for multiple of the TEP peaks (Fig. 15b, more negative in the postperiod). Similar changes in the right M1 were found in response to left M1 TMS (Supplementary Fig. S10). After correction for multiple comparisons (Bonferroni, N = 5), a significant effect of tDCS intensity was found for the peak at*t*= 45 ms (Fig. 15a, more negative with higher tDCS intensity). No tDCS effect was found in the other TEP peaks, even though there were significant post–pre differences around the right M1 area (Fig. 15b). These changes may have been due to motor skill learning or can result from an order effect.

**Fig. 16.**
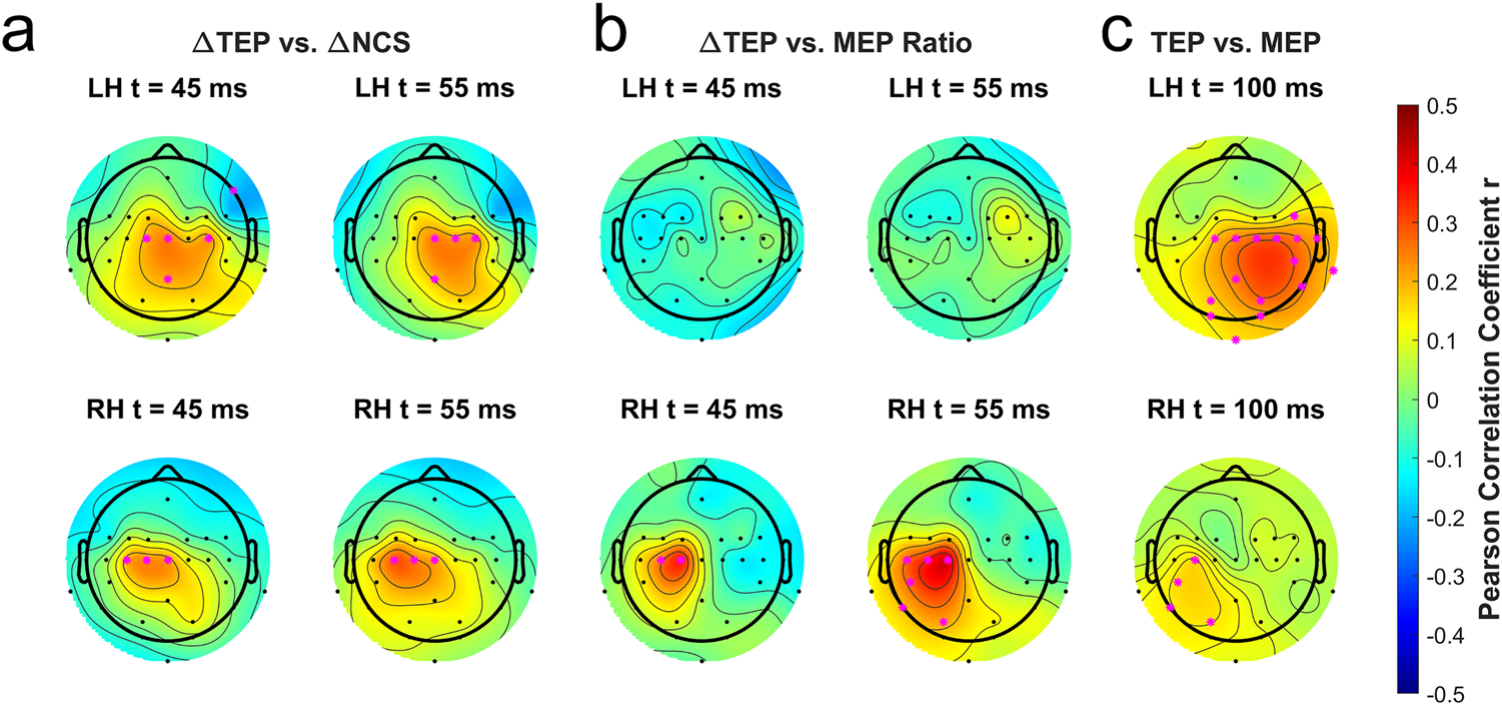
Correlations between TEPs and motor performance and MEPs. Pink asterisks represent channels where a significant correlation was found with*p*< 0.05. The top row shows TEPs from TMS over the right M1, whereas the bottom row shows TEPs from TMS over the left M1. (a) Topographical representations of Pearson correlation coefficients between post–pre changes in TEP amplitude in each channel and motor performance gain at 45 and 55 ms after TMS. The top row performance gains are from the initial task, and the bottom row performance gains are from the follow-up task using the right hand (R:S2). (b) Topographical representations of Pearson correlation coefficients between post–pre changes in TEP amplitude in each channel and post-/pre-MEP amplitude ratios at 45 and 55 ms after TMS. The top row shows MEP ratios in the left hand, and the bottom row shows MEP ratios in the right hand. (c) Topographical representations of Pearson correlation coefficients between TEP amplitudes in each channel and MEP amplitudes, both pre- and poststimulation. The top row shows MEP amplitudes on the left hand, and the bottom row shows MEP amplitudes on the right hand.

To test whether these TEP effects were due to motor learning, we measured correlations between change in TEP amplitudes and motor performance gains. Significant positive Pearson correlations were found between post–pre TEP amplitude changes near M1 and motor performance gain in the contralateral hand at 45 and 55 ms after TMS (Fig. 16a). We fit a linear mixed-effects model with tDCS intensity and motor performance gain as fixed effects and subjects as random effects, but there was no interaction between the effects on the left hand and right M1 at either 45 ms (*F*(1,115) = 2.14,*p*= 0.146) or at 55 ms (*F*(1,115) = 1.81,*p*= 0.182). Likewise, there was no such interaction in the right hand and left M1 (C1 and C3) at either 45 ms (*F*(1,115) = 1.30,*p*= 0.257) or at 55 ms (*F*(1,115) = 0.210,*p*= 0.648). This suggests that the correlation of TEP changes with motor skill learning is not modulated by tDCS. No correlation was found between post–pre change in TEP amplitude and MEP amplitude ratio on the left hand, but there appear to be significant correlations in left M1 channels C1 and C3 for TMS over the left M1 at 45 and 55 ms (Fig. 16b). Nonetheless, there was no interaction between tDCS intensity and MEP ratio at either 45 ms (*F*(1,104) = 0.454,*p*= 0.502) or 55 ms (*F*(1,104) = 0.121,*p*= 0.728). At 100 ms after TMS, there were widespread correlations between TEP amplitudes and MEP amplitudes (both pre- and post-tDCS) in the parietal cortex in the hemisphere where TMS was applied (Fig. 16c). However, these later peaks are likely due to auditory responses to the TMS coil noise (Hernandez-Pavon et al., 2023;ter Braack et al., 2015), or could represent somatosensory feedback.

## 5 Discussion

### 5.1 Summary of results

**Fig. 17.**
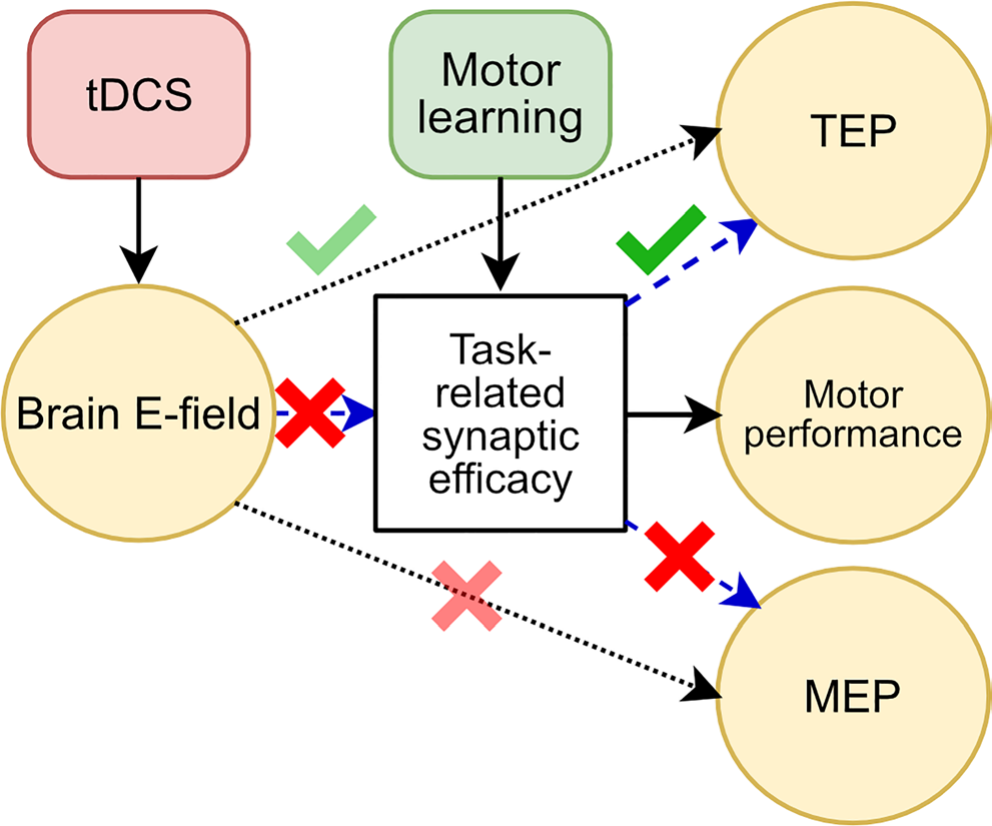
A summary of our findings on tDCS action and interaction with motor skill learning. Black solid lines indicate established causal effects. Dotted lines represent established causal effects that were indirectly tested here. Dashed lines indicate hypothesized causal mechanisms which are less well established. Green check marks indicate current results consistent with hypothesized effects, and red crosses indicate a lack of evidence in support of the hypothesized effects. Lighter shading highlights a caveat, namely that we did not test these effects in the absence of motor learning, that is, during rest, as is usually done.

The purpose of this study was to examine the dose–response effects of high-intensity tDCS on motor sequence learning in terms of behavioral and neurophysiological measures. We also aimed to replicate our previous finding, which demonstrated a significant improvement in motor skill learning with concurrent 4 mA tDCS.Figure 17summarizes our findings in an updated version of our hypothesized effects. Contrary to hypothesis H1, no significant difference in motor performance was observed across tDCS intensities. This suggests that concurrent tDCS at the intensities used here does not have a substantial modulatory effect on the neural substrate underlying motor skill learning. Furthermore, the absence of an effect on MEP refutes hypothesis H2, indicating that under our experimental conditions, tDCS at these intensities does not significantly influence corticospinal excitability. Additionally, we found no correlation between motor performance and change in MEP amplitude, contradicting hypothesis H3, which questions the assumptions that corticospinal excitability is linked to task-related synaptic efficacy.

### 5.2 Significance of results

This study found no effect of 4 or 6 mA tDCS on motor learning, despite our expectation that this protocol would generate field intensities up to 1.8 V/m in the brain. This would have been comparable with previous*in vitro*studies showing a robust boost in synaptic plasticity (Kronberg et al., 2020). There are a number of possible reasons for this lack of an effect. For example, it is not clear that the sequence learning task we used here relies solely on synaptic plasticity in the motor cortex. Several brain regions may be involved that were not optimally targeted with our bipolar configuration (Wischnewski et al., 2024). Future studies may continue to apply HD-tDCS and current flow modeling, taking multiple targets into account. Alternatively, effects may be nonlinear, such that lower intensities (typically 1–2 mA, which we have not tested) are effective, while higher intensities are not. As one of few registered reports on tDCS, we were not able to replicate the effect we previously found with 4 mA (Hsu et al., 2023). However, changes in experimental design may have led to this discrepancy (see below). If future studies can rule out this possible confound, we would have to conclude that our earlier study was a false discovery.

### 5.3 Effects on corticocortical excitability (TEP)

As an exploratory measure, we analyzed changes in TEPs, which have recently garnered interest as neurophysiological correlates of neuroplasticity (Pascual-Leone et al., 2011;Tremblay et al., 2019). We found TEP changes in the stimulated M1 that correlated with motor performance gains. These findings support the notion of a common substrate of motor performance and TEP (green check inFig. 17). In particular, they are consistent with a cortical substrate of skill learning and the argument that M1 is involved in early consolidation of motor learning (Floyer-Lea & Matthews, 2005;Muellbacher et al., 2001,^2002^). Unlike MEPs, there were correlations between TEPs and motor learning gains. It is, therefore, possible that this early stage of motor sequence learning is localized in the cortex rather than the corticospinal tract.

We did observe a change in TEPs in the stimulated M1 that increased with tDCS intensity, pointing to a possible neuromodulatory effect on intracortical plasticity unrelated to learning gains. However, these changes in TEPs were measured with concurrent learning (hence the light-green check mark inFig. 17). It is not clear whether these are the same as the effect of tDCS observed at rest (Ahn & Fröhlich, 2021). Further investigation would be required to test for interactions in a 2 x 2 design involving tDCS and sham, as well as training and no training.

### 5.4 Effects on corticospinal excitability (MEP)

We saw an increase in MEP amplitude across all conditions. This could have been the result of learning, or a simple order effect, such as an experimenter applying TMS differently before and after tDCS and learning. The 2 x 2 design mentioned above could address this confound. Previous studies in the literature did not show an effect of motor sequence learning on MEP amplitudes (Cao et al., 2022;Hirano et al., 2017), although there is evidence of an effect on spinal excitability and plasticity (Lungu et al., 2010;Vahdat et al., 2015). In contrast, there are numerous reports of an effect from ballistic and force-related learning tasks (Christiansen et al., 2018;Classen et al., 1998;Koeneke et al., 2006;Miyaguchi et al., 2019;Muellbacher et al., 2001,^2002^;Rosenkranz et al., 2007;Smyth et al., 2010). Since MEP amplitudes correlated with neither the total number of keypresses during the initial task nor baseline typing speed, we can disregard effects of fatigue from muscle exertion or baseline neurophysiology. An increase in MEP on the untrained hand was also unexpected. Although intermanual transfer of skill learning is well documented in the literature (Dickins et al., 2015;Dirren et al., 2021;Perez et al., 2007;Shea et al., 2011), MEP in the untrained hand does not change following motor learning (Garry et al., 2004;Miyaguchi et al., 2019). This raises our concerns of the abovementioned order effect.

### 5.5 Comparison with our previous results and pilot data

This study does largely parallel the parameters of our own previous study on 4 mA, which showed an effect of tDCS on motor learning (Hsu et al., 2023) as well as the pilot study at 6 mA reported during preregistration, which did not show an effect. The main difference between this and our previous investigation (Hsu et al., 2023) has been the introduction of a pretraining typing task, TMS excitability measurements before tDCS and training, and EEG recordings. It is possible that TMS interacted with tDCS application inducing a homeostatic effect that reduced the hypothesized neuromodulatory influences of tDCS on learning that we and others reported (Jung & Ziemann, 2009;Karabanov et al., 2015). Random group assignments, double blinding, and controlling for baseline performance should have precluded collection of inhomogeneous data sets, increasing the rigor over our previous study. An additional deviation was the use of EEG, for which the gel may have shunted currents through the scalp. If that was the case, the same tDCS montage would have produced weaker field intensities in the motor cortex than expected. Finally, we cannot rule out the possibility that the pretraining typing task interacted with tDCS to reduce its effects on motor learning, although the correlation of typing speed with motor learning observed here makes this less likely.

### 5.6 Comparisons with broader previous findings

Here we provide a brief comparison of experimental conditions and findings from this study and select studies from the literature (Table 2). Our results seem inconsistent with previous reports on the effects of tDCS on MEP in humans (Ahn & Fröhlich, 2021;Ambrus et al., 2016;Mosayebi Samani et al., 2019;Nitsche & Paulus, 2000;Nitsche et al., 2007;Pellicciari et al., 2013), and consistent with previous reports of a lack of an effect on MEP (Jonker et al., 2021;Pillen et al., 2022;Wiltshire & Watkins, 2020). They also seem to conflict with reported effects on motor skill learning in humans (Galea & Celnik, 2009;Iannone et al., 2022;Nitsche et al., 2003;Saucedo Marquez et al., 2013;Stagg, Jayaram, et al., 2011), but are consistent with others that show no effect (Ambrus et al., 2016;Nguemeni et al., 2021;Wiltshire & Watkins, 2020). We emphasize that these outcomes are specific to our configuration of stimulation montage, target, dose, training task, measurements, etc. Therefore, the difference from the literature may be the results of these experimental choices. For instance, it is important to note that we cannot rule that concurrent learning may have interfered with the effects of tDCS on MEP previously observed during rest (Ahn & Fröhlich, 2021;Mosayebi Samani et al., 2019;Nitsche & Paulus, 2000;Nitsche et al., 2007), but seeAmbrus et al. (2016),Antal et al. (2007),Pellicciari et al. (2013). Other factors that differed were electrode montage, the use of the dominant versus nondominant hand, and a possible interaction of TMS with tDCS. A higher than usual number of TMS pulses were applied here in order to measure TEPs (Hernandez-Pavon et al., 2023), although this is not unprecedented for tDCS studies (Ahn & Fröhlich, 2021;Mosayebi-Samani et al., 2023;Pellicciari et al., 2013). Additionally, it is possible that behavioral effects of tDCS related to learning are not observable in such a short period or within one session, as post-training consolidation is another crucial phase of learning.

**Table 2.** Comparison of tDCS protocols and outcomes from select studies.

Study	Montage	Dose	Task	Measurement	Consistent
This study	Right M1 HD-tDCS	4–6 mA, 12 min	FTT	Behavior, MEP, TEP	N/A
Hsu et al., 2023	Right M1 HD-tDCS	4 mA, 12 min	FTT	Behavior	No
6 mA pilot	Right M1 HD-tDCS	6 mA, 12 min	FTT	Behavior	Yes
Nitsche and Paulus, 2000	Left M1-SO	1 mA, 4 s	None	MEP	No
Pillen et al., 2022	Left M1-SO	1 mA, 4 s	None	MEP	Yes
Ahn and Fröhlich, 2021	Left M1-SO	2 mA, 11 min	None	MEP, TEP	No
Jamil et al., 2017	Left M1-SO	0.5–2 mA, 15 min	None	MEP	No
Reis et al., 2009	Left M1-SO	1 mA, 20 min	SVIPT, 5 days	Behavior	No
Reis et al., 2015	Left M1-SO	1 mA, 20 min	SVIPT, 3 days	Behavior	No
Ambrus et al., 2016	Left M1-SO	1 mA, 12–14 min	SRTT	Behavior, MEP	Yes
Pellicciari et al., 2013	Left M1-SO	1 mA, 13 min	SRTT-like	Behavior, MEP, TEP	No
Nitsche et al., 2003	Left M1-SO	1 mA, 15 min	SRTT	Behavior	No
Saucedo Marquez et al., 2013	Right M1-left shoulder	1 mA, 20 min	FTT	Behavior	No
Shinde et al., 2021	Right M1-SO or M1-M1	2–4 mA, 10 min	FTT	Behavior, rCBF	No

Last column indicates which results are consistent with the results of the current study.

### 5.7 Nonlinear effects and sensation effects

Our basic hypothesis that higher intensities should cause larger learning effects was based on*in vitro*studies with synaptic plasticity (Sharma et al., 2022). However, neuroplasticity underlying*in vivo*motor skill learning in humans may be quite different, spanning multiple brain regions throughout different stages of acquisition and consolidation (Dayan & Cohen, 2011). Due to these differences in complexity, dose effects may not reproduce similarly. Contrary to our hypotheses, there may have been inhibitory or reversing effects of tDCS alluded to in the literature, albeit not at such high intensities (Batsikadze et al., 2013;Hassanzahraee et al., 2020). We did not find a sensation effect or an interaction effect between current intensity and sensation on motor performance. This argues against sensation as a detrimental effect that could have impeded performance gains. Due to the opposing effects of tDCS with opposing polarity (Agboada et al., 2019;Batsikadze et al., 2013;Kronberg et al., 2020;Lafon et al., 2017;Mosayebi Samani et al., 2019) yet similar sensation, it may be worthwhile to test the effects of cathodal stimulation at these intensities in a future study.

### 5.8 Anterior-to-posterior tDCS montage

We used an electrode montage with currents flowing from posterior to anterior direction with electrodes straddling M1. Similar to other studies targeting M1 (Rawji et al., 2018;Wischnewski et al., 2024), the goal was to depolarize the motor strip on the anterior of the central sulcus (Hsu et al., 2023). However, the electric field distribution for this configuration is quite different from the typical M1-SO configuration used in most previous tDCS studies (Hsu et al., 2023). Therefore, while we maximized intensity orthogonal to the cortical surface of M1, stimulation of other cortical structures may have been suboptimal. For example, in optimizing the stimulation montage for electric field intensity in a specific orientation (Dmochowski et al., 2013;Evans et al., 2022;Huang et al., 2019;Rahman et al., 2013;Rawji et al., 2018), we sacrificed focality (DaSilva et al., 2015;Dmochowski et al., 2011;Huang & Parra, 2019). Because the electric fields were quite diffuse in our configuration (Hsu et al., 2023), it is possible that polarization of nontarget areas inhibited learning-related plasticity, especially when field intensities under 6 mA were estimated to be as high as 1.8 V/m (see Pilot Data below). For example, due to opposite cortical surface orientations, a current that depolarizes M1 could hyperpolarize the homologous somatosensory cortex on the posterior wall of the central sulcus, which is thought to be recruited during motor skill learning (Borich et al., 2015;Honda et al., 1998;Vidoni et al., 2010), leading to opposite effects. Similarly, the premotor cortex may have been stimulated with the opposite polarity. Yet, the premotor cortex has an excitatory connection with the primary motor cortex (Wischnewski et al., 2024). Indeed, a study with anterior-to-posterior tDCS currents demonstrated a suppression of MEP (Rawji et al., 2018). Finally, it is evident that tDCS outcomes can vary significantly across subjects and may be improved by individualized electrode montages and dosages (Datta et al., 2012;Evans et al., 2022;Huang et al., 2017;Laakso et al., 2019,^2023^;Liu et al., 2018), while we instead prioritized efficiency, replicability, and statistical power by implementing the same montage for all subjects.

### 5.9 Caveats

Except for a short typing task, the present study was identical to our previous study (Hsu et al., 2023) in terms of behavioral demands to the subject. Nevertheless, the procedures added about 60 min at the start with the participant at rest. Although the pre-tDCS and learning task procedures were not physically demanding, there could have been cognitive fatigue, especially from TMS. Additionally, the TMS during the pause could have in theory affected task performance. An overall improvement in performance between the initial 12-min training session and the follow-up tasks after a 60-min pause indicates that subjects were still attentive at that point. The measurement of effects on subsequent training was exploratory as this study was not designed to test for lasting effects or carryover effects. A failure to replicate our previous results on this may have been a consequence of the intervening TMS or cognitive fatigue.

We were not aware of reports on lasting effects of 0.2 Hz single-pulse TMS, despite decades of research using this modality. We, therefore, did not expect any interactions with the sequence learning task nor tDCS. At the same time, we cannot in theory rule out such interaction effects, as the literature has not yet seen TMS combined with the intensity levels of tDCS used here.

We considered the possibility that the combination of tDCS with motor learning may saturate excitability. Because significant changes in both MEP and TEP were observed after learning in the control group, saturation in the active groups cannot be ruled out.

Despite applying more TMS pulses than typically done for MEP measurements and ensuring precise coil placement with neuronavigation, we observed high variance in MEP amplitudes both across trials and across subjects, although not anomalous to previous studies (Agboada et al., 2019;Amadi et al., 2015;Jamil et al., 2017;Jonker et al., 2021). Possible contributing factors to this variance include cognitive fatigue in the participant, physical and/or cognitive fatigue in the experimenter, and acoustic effects of TMS. Results may be improved by optimizing the number of stimuli, further training of experimenters, or masking the TMS coil sound (Hernandez-Pavon et al., 2023;Nikouline et al., 1999;ter Braack et al., 2015).

## 6 Conclusions

In conclusion, under the experimental conditions tested here, tDCS at 4 and 6 mA does not appear to modulate motor learning performance or corticospinal excitability, and these two do not appear to share a common neural substrate. Our previous findings of an effect of 4 mA tDCS on motor learning were not replicated here with a large sample size, highlighting the importance of rigorous control measures in experimental design. One caveat is that the addition of TMS may have interfered with the effects of tDCS. There are signs of learning-related plasticity in TEPs that warrant further investigation as potential targets of neuroplasticity. Given the breadth of the tDCS parameter space, further improvements in stimulation techniques as well as motor learning paradigms may be necessary to yield replicable behavioral and neurophysiological outcomes.

## Study Timeline

2023/10/06  Study preregistration on OSF

2023/10/09  Data collection begins

   Blinded quality control in parallel with data collection

   Blinded coding for processing and analysis

2024/01/11  Submission of report to PCI RR (version 1.0)

2024/01/30  Submission of report to PCI RR (version 1.1)

2024/04/09  Submission of report to PCI RR (version 1.2)

2024/05/22  Submission of report to PCI RR (version 1.3)

2024/05/23  Completion of data collection

2024/05/24  Data processing/analysis and unblinding

2024/06/05  In-principle acceptance of manuscript by PCI RR

2024/09/02  Submission of Stage 2 report to PCI RR (version 2.0)

2024/10/25  Submission of Stage 2 report to PCI RR (version 2.1)

2024/11/08  Final recommendation by PCI RR

## Supplementary Material

Supplementary Material

## Data Availability

Deidentified data and analysis code are available at:https://doi.org/10.17605/OSF.IO/GYFAE
