# Supplementary material for: Dose–response of tDCS effects on motor learning and cortical excitability: A preregistered study
Source: Imaging Neurosci (Camb). 2025 Jan 15;3:imag_a_00431. doi: 10.1162/imag_a_00431 (PMC12319989; doi:10.1162/imag_a_00431)
Supplement: Supplementary Material [file imag_a_00431-supp.pdf]

Supplementary Material

Validity of NCS as a measure of speed and accuracy

The average NCS throughout the initial learning task was nearly perfectly correlated with average speed throughout the task (Fig. S1a). It was also weakly correlated with average accuracy across trials (number of correct keypresses divided by total number of keypresses, Fig. S1b), whereas speed was not correlated with accuracy (Fig. S1c). Thus, we demonstrate that counting the number of correct sequences in each trial is a valid measure of motor performance that accounts for both speed and accuracy. We also find a strong correlation between average NCS and the change in NCS from the first trial to the last 10 trials (Fig. S1d), which shows that there was no ceiling effect, as even strong performers improved throughout the task.

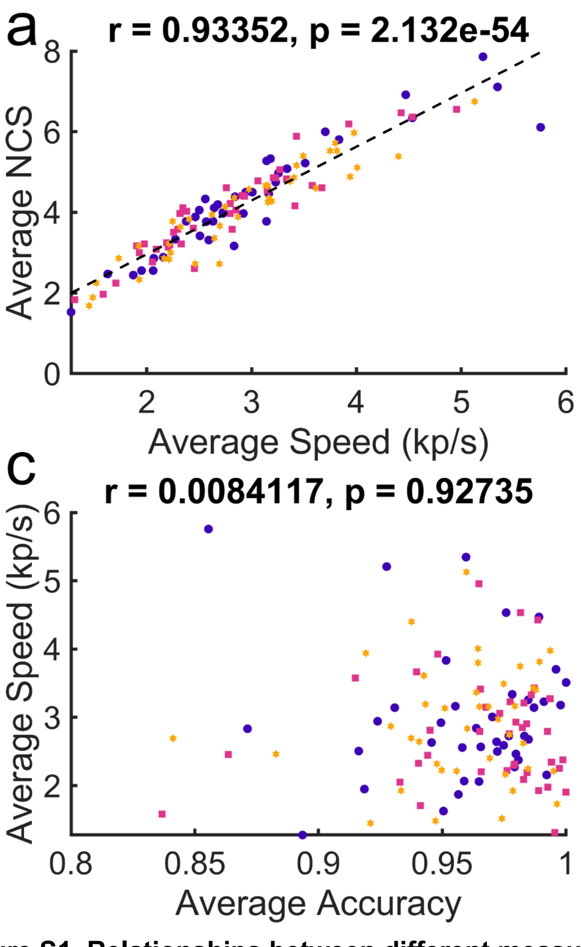

Figure S1. Relationships between different measurements of motor performance. Points represent individual subjects and dashed lines show linear regressions. **(a)** Average NCS vs. average typing speed throughout the initial task. **(b)** Average NCS vs. average accuracy. **(c)** Average tapping speed vs. average accuracy. **(d)** Average NCS vs. change in NCS from first trial to last 10 trials.

Other effects on motor performance

We find in a two-sample t-test that subjects who participated in the morning sessions had significantly higher typing speed than those who participated in the afternoon (t(118) = 2.04, p = 0.0437, Fig. S2a), even though typing speed was counterbalanced across groups. However, this was not linked to a significant difference in motor performance during the actual learning task (t(118) = 0.829, p = 0.409, Fig. S2b). There was no difference by participant sex in typing speed (t(118) = 0.330, p = 0.742, Fig. S2c) or motor performance (t(118) = 1.46, p = 0.148, Fig. S2d). There was a negative correlation between typing speed and age (r(118) = -0.241, p = -0.0081, Fig. S2e) but not between motor performance and age (r(118) = -0.0231, p = 0.802, Fig. S2f).

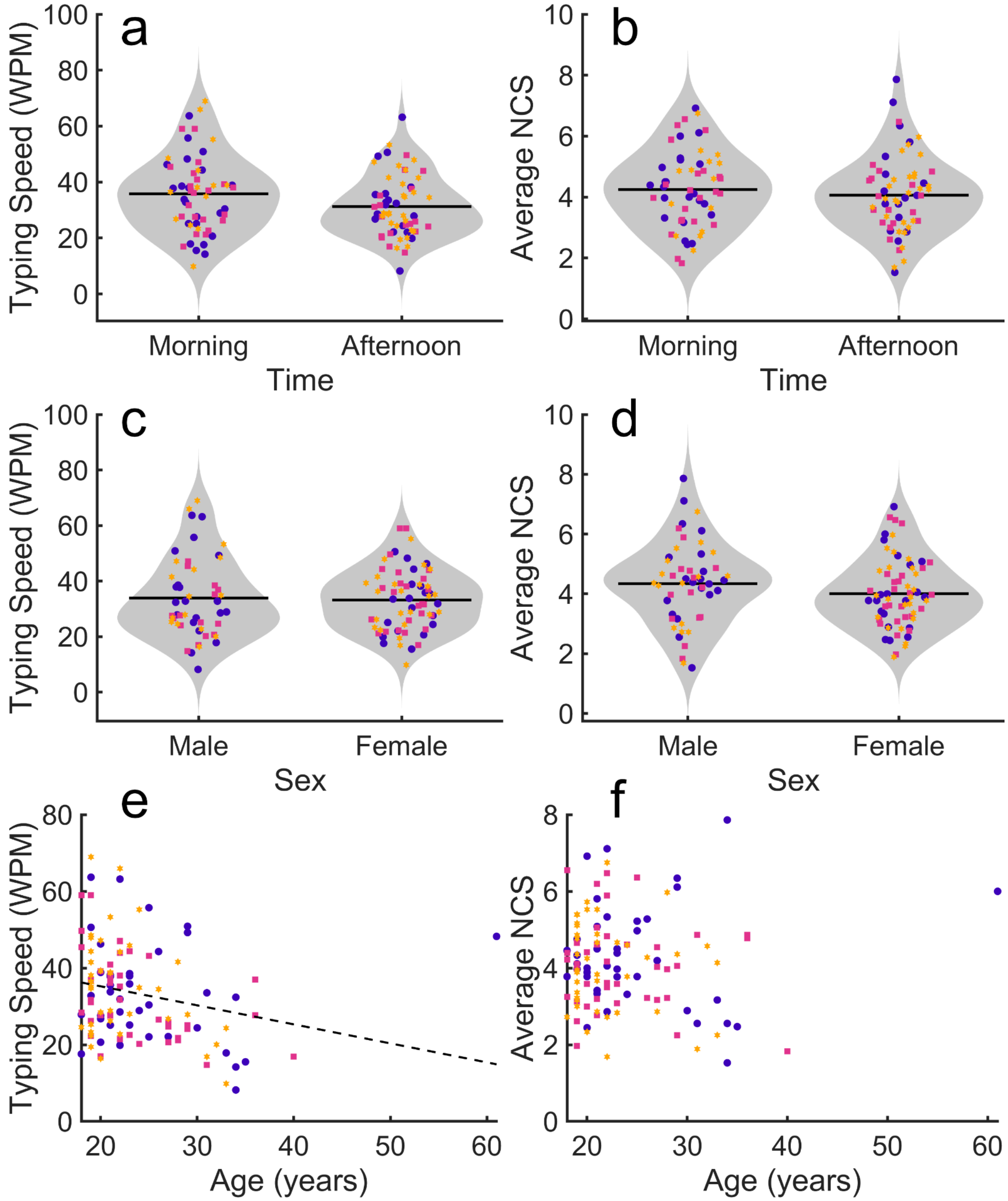

Figure S2. Comparisons of typing speed and motor performance based on time of day and participant demographics. Points represent individual subjects, horizontal bars represent means, and shaded areas represent kernel density estimates. **(a)** Typing speed vs. time of day. **(b)** Average NCS vs. time of day. **(c)** Typing speed vs. sex. **(d)** Average NCS vs. sex. **(e)** Typing speed vs. age. Dashed line shows linear regression. **(f)** Average NCS vs. age.

Other performance metrics

All subjects included in our analyses exhibited positive learning gains ΔNCS, calculated as the difference between NCS averaged across the last 10 trials and NCS averaged across the first trial (Fig. S3). There was no dose response effect of tDCS on ΔNCS.

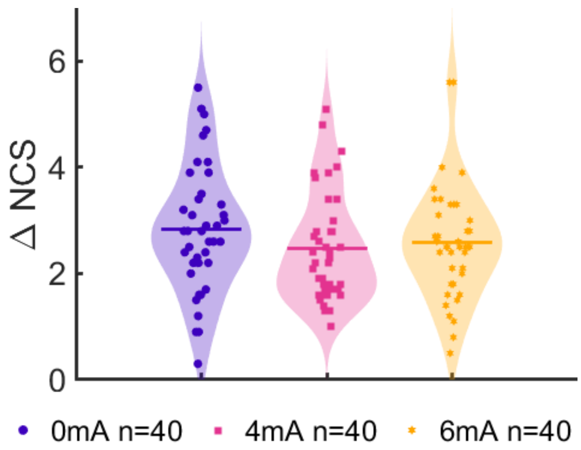

Figure S3. Motor learning gains. Points represent individual subjects, bars represent means, and shaded areas represent kernel density estimates. Motor performance gain is calculated as the difference between NCS in the last 10 trials and the first trial.

Effects of neuromuscular fatigue and typing speed on MEP

Post/pre MEP ratio did not correlate with the total number of keypresses throughout the initial motor learning task (Fig. S4a). This could mean that changes in corticospinal excitability were not negatively affected by neuromuscular fatigue from repeated muscle movements, or that more exertion did not lead to higher excitability. Additionally, the lack of a correlation between change in MEP and typing speed (Fig. S4b) indicates that stronger baseline motor performance is not linked to corticospinal excitability. This also shows that muscle exertion during the typing test likely did not confound MEP results.

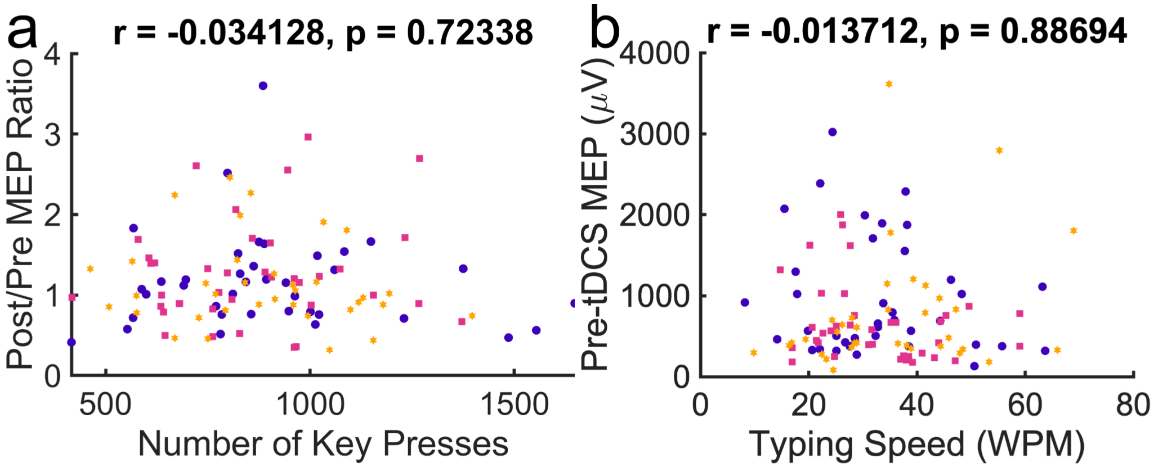

Figure S4. Relationships between repeated finger movements and change in MEP amplitude. Points represent individual subjects. **(a)** Post/pre MEP amplitude ratio vs. total number of keypresses throughout the initial task concurrent with tDCS. **(b)** Pre-tDCS MEP amplitude vs. typing speed.

Effects on MEP in the unstimulated hand

MEP amplitudes increased overall after tDCS and learning (Fig. S5). A linear model finds an intercept of 1.44 ($t(109) = 12.8$, $p = 3.32 \times 10^{-23}$, SEM = 0.0651).

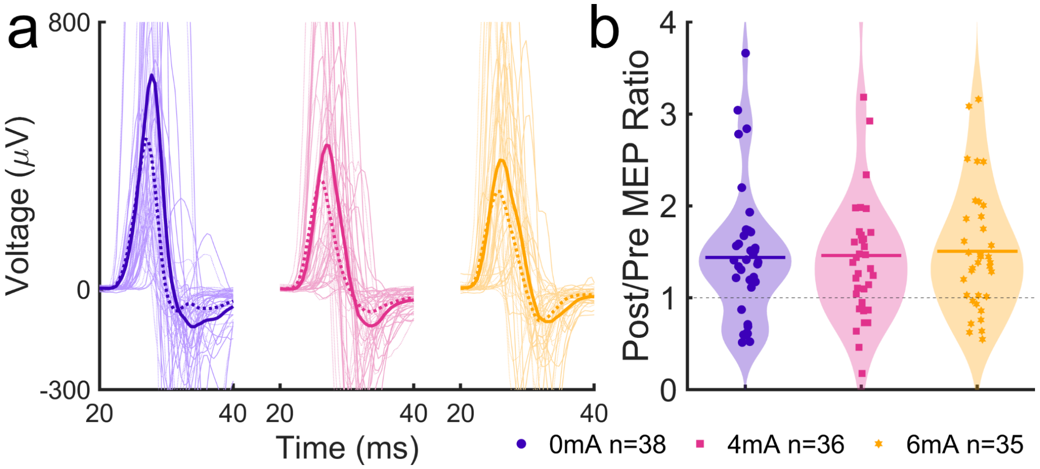

Figure S5. Change in MEP amplitude in the unstimulated hand following different stimulation conditions. (a) Pre- and post-tDCS MEP recordings, epoched around time = 0 at the TMS trigger. Thin lines represent median MEPs across trials for individual subjects and bold lines represent mean MEPs across subjects within groups. Dotted lines represent pre-stimulation and solid lines represent post-stimulation. **(b)** Post/pre-stimulation MEP amplitude ratios. Points represent individual subjects, bars represent within-group means, and shaded areas represent kernel density estimates.

Initial and saturated performance

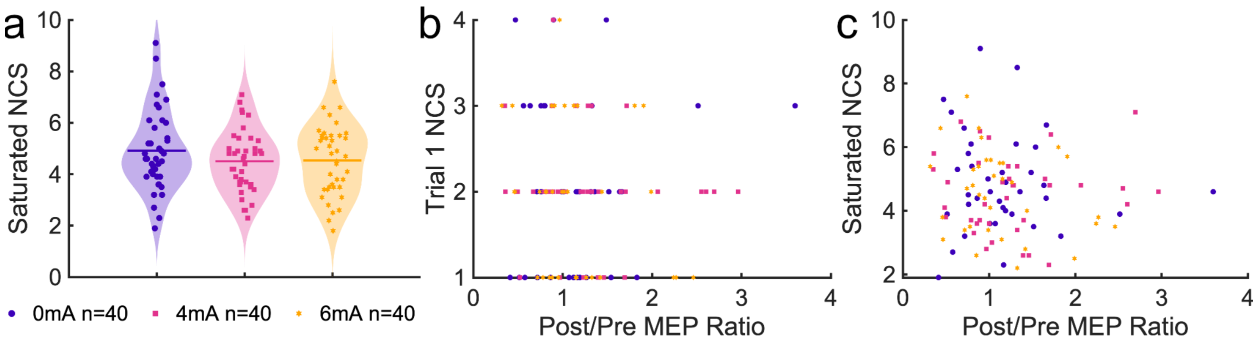

Figure S6. Alternative measures of behavioral performance. Points represent individual subjects, bars represent within-group means, and shaded areas represent kernel density estimates. **(a)** NCS averaged for the last 10 trials (saturated NCS) in the different tDCS groups. **(b)** Scatter plot of NCS at the start of the experiment (Trial 1) and MEP ratio. **(c)** Scatter plot of saturated NCS and MEP ratio.

Sensation Quality Ratings

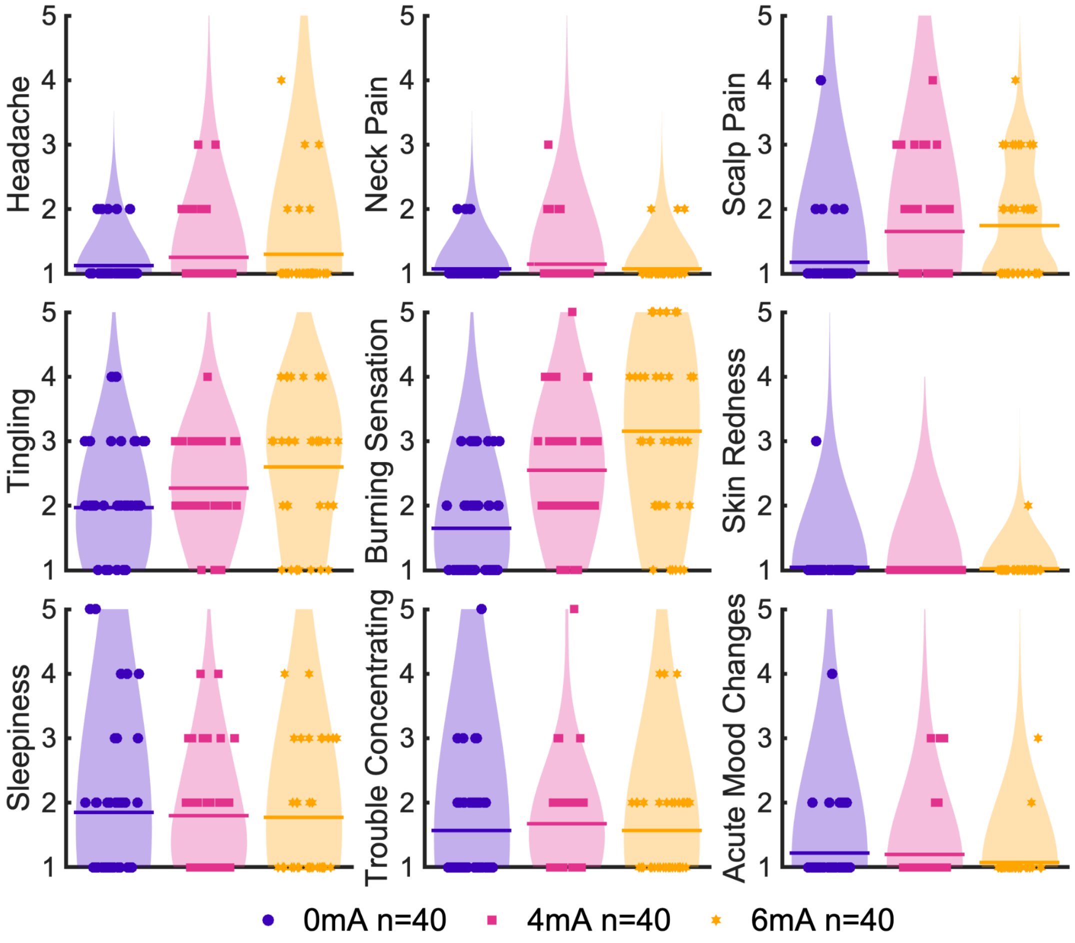

Figure S7. Severity ratings of different sensation qualities of tDCS, collected in an adverse event reporting questionnaire as secondary safety outcomes. Ratings were discrete integer values from 1 to 5, corresponding to the following descriptions: “Absent”, “Mild”, “Moderate”, “Severe”, and “Extreme”. Points represent individual subjects, bars represent within-group means, and shaded areas represent kernel density estimates.

Performance Speed

Motor performance may alternatively be measured simply as reaction time of muscle movements. Here we calculate finger tapping speed during each trial by counting the number of keypresses per second in completely correct sequences and any correct but incomplete sequences at the end of the trials, as described by Bönstrup et al., 2019. We find no dose effect of tDCS on speed in any of the tasks (Fig. S8).

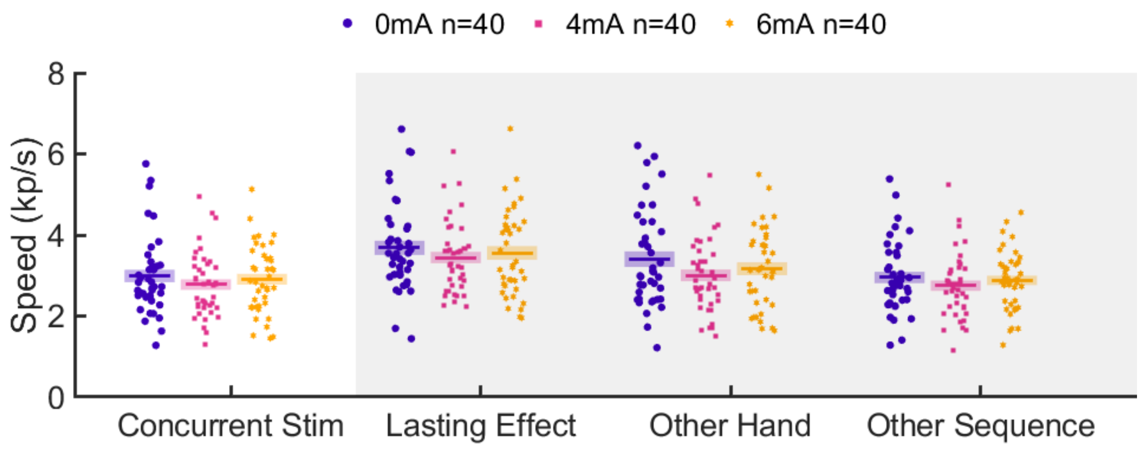

Figure S8. Performance measured as speed. Points represent individual subjects, horizontal bars represent within-group means, and colored shaded areas represent SEM. The initial learning task was performed concurrently with tDCS, using the left hand and training on sequence S1 (L:S1). The shaded area denotes follow-up tasks performed 1 hour after the end of the initial task and tDCS, in the order shown here from left to right. First, the same trained sequence S1 was repeated on the left hand, followed by a new sequence S2 on the right hand, and finally a new sequence S3 was trained on the left hand.

Effects on TEP in the unstimulated hemisphere

TEPs for TMS applied to the left M1 and right hand (Fig. S9) appear similar to those applied on the contralateral side (Fig. 9). Deflections occur at approximately the same time (panels a) and the spatial pattern (panels b) seem to mirror that on the opposite side. No significant differences across groups were found in the post-pre changes in TEP amplitude in C2 and C4 (Fig. S10a), even though there were overall post-pre changes in C2 and C4 across all conditions (Fig. S10b).

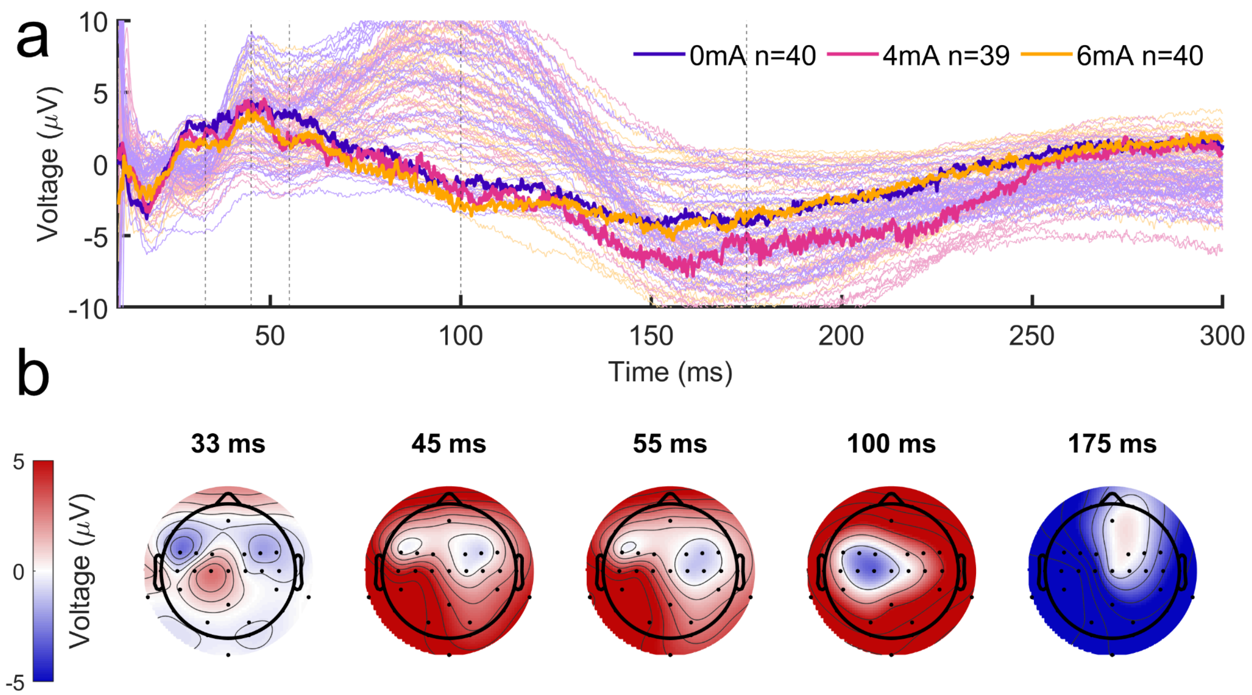

Figure S9. Time course of TEPs following stimulation of left M1 and right hand. (a) Thin lines represent pre-stimulation TEPs from individual channels, averaged across subjects within each group. Bold lines represent median pre-stimulation TEPs across subjects within each group, averaged across right M1 channels C2 and C4. **(b)** Topographical representations of TEP peaks corresponding to conventionally reported TEP components from the literature (P30, N45, P60, N100, and P180).

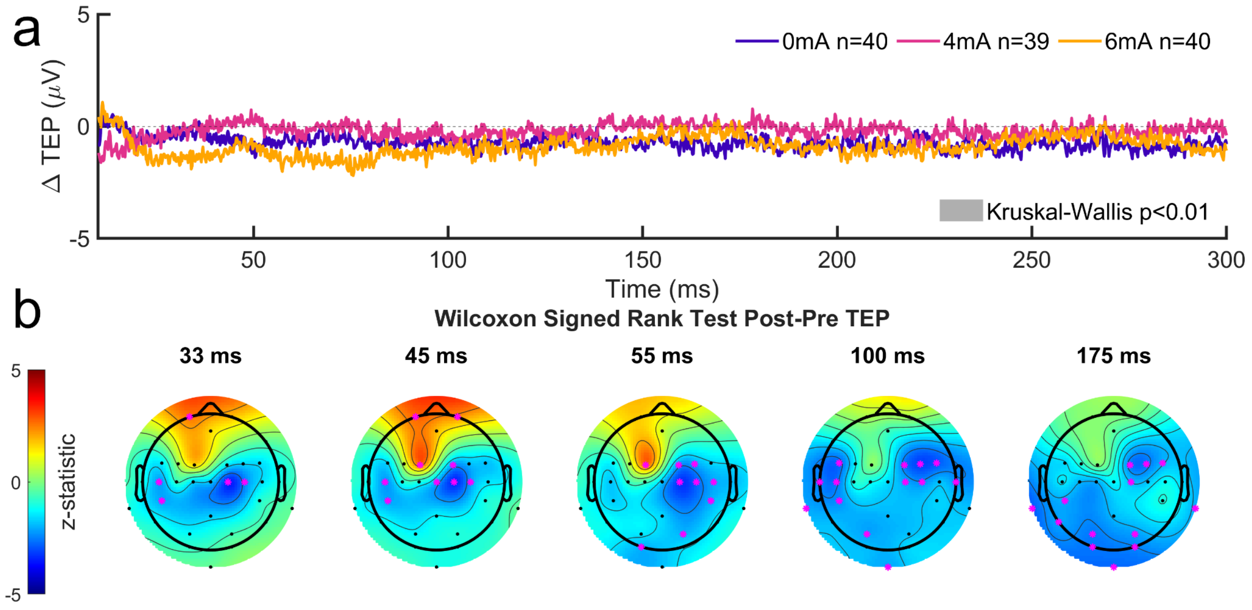

Figure S10. Post-Pre changes in TEPs following stimulation of left M1 and right hand. (a) Median post-pre difference in TEPs across subjects within each group, averaged across right M1 channels C2 and C4. Shaded areas represent time points where TEPs averaged over a 10-ms window around the time

point were significantly different across groups ($p < 0.05$) in a Kruskal-Wallis test. **(b)** Topographical representations of z-statistics from Wilcoxon signed rank tests comparing pre- and post- stimulation TEP amplitudes averaged over 10-ms windows around peak times corresponding to conventionally reported TEP components from the literature (P30, N45, P60, N100, and P180). Pink asterisks represent channels where a significant difference was found ($p < 0.05$).
